# Continued Decay of HIV Proviral DNA Upon Vaccination With HIV-1 Tat of Subjects on Long-Term ART: An 8-Year Follow-Up Study

**DOI:** 10.3389/fimmu.2019.00233

**Published:** 2019-02-13

**Authors:** Cecilia Sgadari, Paolo Monini, Antonella Tripiciano, Orietta Picconi, Anna Casabianca, Chiara Orlandi, Sonia Moretti, Vittorio Francavilla, Angela Arancio, Giovanni Paniccia, Massimo Campagna, Stefania Bellino, Marianna Meschiari, Silvia Nozza, Laura Sighinolfi, Alessandra Latini, Antonio Muscatello, Annalisa Saracino, Massimo Di Pietro, Massimo Galli, Aurelio Cafaro, Mauro Magnani, Fabrizio Ensoli, Barbara Ensoli

**Affiliations:** ^1^National HIV/AIDS Research Center, Istituto Superiore di Sanità, Rome, Italy; ^2^Department of Biomolecular Science, University of Urbino, Urbino, Italy; ^3^Division of Infectious Diseases, University Policlinic of Modena, Modena, Italy; ^4^Division of Infectious Diseases, San Raffaele Hospital, Milan, Italy; ^5^Unit of Infectious Diseases, University Hospital of Ferrara, Ferrara, Italy; ^6^Unit of Dermatology and Sexually Transmitted Diseases, San Gallicano Dermatological Institute IRCCS, Rome, Italy; ^7^Infectious Diseases Unit, IRCCS Ca' Granda Ospedale Maggiore Policlinico Foundation, University of Milan, Milan, Italy; ^8^Division of Infectious Diseases, University of Bari, Policlinic Hospital, Bari, Italy; ^9^Unit of Infectious Diseases, Santa Maria Annunziata Hospital, Florence, Italy; ^10^Institute of Tropical and Infectious Diseases, L. Sacco Hospital, University of Milan, Milan, Italy; ^11^Pathology and Microbiology, San Gallicano Dermatological Institute IRCCS, Rome, Italy

**Keywords:** HIV therapeutic vaccine, Tat, cART, anti-Tat antibodies, CD4^+^ T cells, CD4^+^/CD8^+^ T-cell ratio, proviral DNA, HIV reservoirs

## Abstract

**Introduction:** Tat, a key HIV virulence protein, has been targeted for the development of a therapeutic vaccine aimed at cART intensification. Results from phase II clinical trials in Italy (*ISS T-002*) and South Africa (*ISS T-003*) indicated that Tat vaccination promotes increases of CD4^+^ T-cells and return to immune homeostasis while reducing the virus reservoir in chronically cART-treated patients. Here we present data of 92 vaccinees (59% of total vaccinees) enrolled in the ISS T-002 8-year extended follow-up study *(ISS T-002 EF-UP, ClinicalTrials.gov NCT02118168*).

**Results:** Anti-Tat antibodies (Abs) induced upon vaccination persisted for the entire follow-up in 34/92 (37%) vaccinees, particularly when all 3 Ab classes (A/G/M) were present (66% of vaccinees), as most frequently observed with Tat 30 μg regimens. CD4^+^ T cells increased above study-entry levels reaching a stable plateau at year 5 post-vaccination, with the highest increase (165 cells/μL) in the Tat 30 μg, 3 × regimen. CD4^+^ T-cell increase occurred even in subjects with CD4^+^ nadir ≤ 250 cells/*u*L and in poor immunological responders and was associated with a concomitant increase of the CD4^+^/CD8^+^ T-cell ratio, a prognostic marker of morbidity/mortality inversely related to HIV reservoir size. Proviral DNA load decreased over time, with a half-life of 2 years and an estimated 90% reduction at year 8 in the Tat 30 μg, 3 × group. In multivariate analysis the kinetic and amplitude of both CD4^+^ T-cell increase and proviral DNA reduction were fastest and highest in subjects with all 3 anti-Tat Ab classes and in the 30 μg, 3 × group, irrespective of drug regimens (NNRTI/NRTI vs. PI). HIV proviral DNA changes from baseline were inversely related to CD4^+^/CD8^+^ T-cell ratio and CD4^+^ T-cell changes, and directly related to the changes of CD8^+^ T cells. Further, HIV DNA decay kinetics were inversely related to the frequency and levels of intermittent viremia. Finally, Tat vaccination was similarly effective irrespective of the individual immunological status or HIV reservoir size at study entry.

**Conclusions:** Tat immunization induces progressive immune restoration and reduction of virus reservoirs above levels reached with long-term cART, and may represent an optimal vaccine candidate for cART intensification toward HIV reservoirs depletion, functional cure, and eradication strategies.

## Introduction

The HIV-1 Tat protein plays essential roles in the virus life cycle and in pathogenesis (1–9). Tat is produced very early upon infection (1–5), is released extracellularly (1, 4, 5) and accumulates in tissues (4) where it exerts effects on both the virus and the immune system (1–11), making it an optimal candidate for therapeutic immunization and combined antiretroviral therapy (cART) intensification (12–15). In particular, extracellular Tat activates virus and cellular gene expression and replication, contributing to disease maintenance under cART (1, 6–9, 16–18). Further, extracellular Tat binds HIV envelope (Env) spikes forming a virus entry complex that favors infection of dendritic cells (DC) and transmission to T cells, key components of the virus reservoir (17). This occurs by redirecting virus entry from the canonical C-type lectin receptors to RGD-binding integrins that Tat uses as receptors to enter DC and other cells of the reticular-endothelial cell system (17). By this mean, Tat enables HIV to enter DC even in the presence of antibodies (Abs) blocking the interaction of Env with C-type lectin receptors, rendering neutralizing Abs ineffective (17). Virus neutralization, however, can be restored and further increased by anti-Tat Abs, induced by either natural infection or vaccination (17). Notably, in natural HIV infection anti-Tat Abs are found only in a small fraction of individuals (19), while in contrast, Abs against all other viral products are present at high titers (20). When present, anti-Tat Abs correlate with the asymptomatic state and lower disease progression (21–24). This suggested that targeting Tat represents a pathogenesis-driven intervention to intensify cART efficacy.

Although cART has radically changed the quality and expectancy of life of HIV-infected individuals, it is unable to fully restore the immune system and is ineffective on the viral reservoirs (25, 26). Moreover, even under fully successful therapy, viral gene products are still produced (18, 27). As a result, chronic inflammation and immune dysregulation persist, leading to a much higher risk of co-morbidities and death as compared to the general population (28), particularly in patients starting cART with very low CD4^+^ T-cell counts (25, 29) or poorly compliant with therapy. Furthermore, cumulative effects of drug toxicity cannot be mitigated through structured drug-free periods, in fact, virus rebound occurs within few weeks upon therapy interruption owing to persistency of viral reservoirs during cART (30). There is, therefore, a great need of novel HIV/AIDS treatments to offset ART shortfalls and to intensify treatment outcomes. A therapeutic HIV vaccine, in conjunction with existing strategies, may represent a relevant, cost-effective contribution to increase ART effectiveness by i) attaining a faster/more effective response to therapy (ART intensification), ii) mitigating the effect of poor adherence to ART, and by iii) reducing virus reservoirs.

After the successful completion of randomized, placebo-controlled, phase I trials with the biologically active HIV-1 Tat protein (31, 32), an “exploratory” multicenter, randomized, open label phase II therapeutic trial was conducted in Italy in 168 anti-Tat Ab negative, virologically suppressed patients on cART since a mean of 6 years (*ISS T-002, Clinicaltrials.gov NCT00751595*) to evaluate the immunogenicity (primary endpoint) and safety (secondary endpoint) of two Tat protein doses (7.5 or 30 μg) administered intradermally 3 times (3 ×) or 5 ×, 1 month apart. All four vaccine regimens were safe and well-tolerated, and induced anti-Tat Abs in most patients (79%), with the highest frequency, titers, breath and durability in the Tat 30 μg groups (89% responders), particularly when given 3 × (92% responders) (15, 33). Most importantly, Tat immunization promoted a durable and significant increase of T, B, natural killer (NK) cells, CD4^+^ and CD8^+^ central memory subsets and of adaptive T-cell responses, with a decrease of immune activation and effector memory T-cell subsets (15, 33). Moreover, a significant increase of NK cells and CD38^+^HLA-DR^+^CD8^+^ T-cells, a phenotype associated with increased killing activity in elite controllers, was observed in the Tat 30 μg, 3 × group (33). Of note, more immune-compromised individuals experienced greater therapeutic effects. Finally, a reduction of proviral DNA was seen after week 72, particularly under HIV protease inhibitors (PI)-based regimens and with Tat 30 μg, 3 ×. This decay was significantly associated with anti-Tat Ab titers and neutralization of Tat-mediated entry of oligomeric Env in DC (33), which predicted at 48 weeks the significant HIV-1 DNA reduction observed since year 3 post-vaccination. None of these changes were observed in subjects on effective cART enrolled in a parallel observational study (ISS OBS T-002) used as reference control group (ClinicalTrials.gov NCT01024556) (15, 33), according to the International Conference on Harmonization (ICH) of Technical Requirements for Registration of Pharmaceuticals for Human Use regulations (34).

A randomized, double-blind, placebo-controlled phase II clinical trial conducted in South Africa confirmed the previous phase II exploratory study conducted in Italy in terms of safety, and induction of durable, high titers anti-Tat Abs of different classes capable of cross-clade recognition and neutralization (35). The study results also confirmed the significant increases of CD4^+^ T cells, above cART therapeutic levels, particularly in subjects with low CD4^+^ T-cell counts at baseline (poor immunological responders) (35), while proviral DNA is currently under analysis in a follow-up study.

The ISS T-002 clinical trial in Italy was completed at 48 weeks; however, at study closure follow-up data were available up to 96 weeks for 76 volunteers, and up to 144 weeks for 45 volunteers. To further extend the follow-up, an observational study (*ISS T-002 EF-UP, ClinicalTrials.gov NCT02118168*) was conducted to evaluate the persistence of anti-Tat immune response and to monitor immunological and virological parameters, including CD4^+^ T-cell counts and HIV proviral DNA load in vaccinees. Here we present the results of immunological and virological disease biomarkers monitored up to 8 years after first vaccination.

## Materials and Methods

### Study Description

The ISS T-002 EF-UP (ClinicalTrials.gov NCT01024556) was an observational study to prospectively follow up the patients enrolled in the ISS T-002 clinical trial (ClinicalTrials.gov NCT02118168) (15, 33). Ninety-two volunteers (59% of the total vaccinees evaluated at trial end), previously immunized intradermally with the biologically active Tat at 7.5 or 30 μg doses (in the absence of adjuvants) given 3 × or 5 × 1 month apart, as by the ISS T-002 study protocol (15), were followed up for a total of 132 weeks. The ISS T-002 EF-UP study started on September 2013 and was closed on July 2016, when the participants had a median follow-up of 120 weeks (range 72–132). The primary endpoint of this study was to evaluate the persistence of the anti-Tat humoral immune response in vaccinees who had received at least 3 immunizations. Evaluations of immunological and virological disease biomarkers, including assessment of the immune recovery and virus reservoirs, were the secondary endpoints. Clinical and routine laboratory evaluations performed at each clinical site were also recorded. The immunological and virological assessments were scheduled about every 3 months, according to the timing of routine clinical monitoring, or, in the absence of signs of disease progression, every 6 months. The study was conducted in 8 Italian clinical sites (Policlinic of Modena, Modena; Arcispedale S. Anna, Ferrara; San Gallicano Dermatological Institute IRCCS, Rome; Policlinic of Bari, Bari; S. Raffaele Hospital, Milan; S. Maria Annunziata Hospital, Florence; Luigi Sacco Hospital, Milan; San Gerardo Hospital, Monza). All subjects signed an informed consent prior to enrollment. The study protocol was submitted and approved by the competent authorities (Director General of the Coordinating Clinical Center, S. Maria Annunziata Hospital, Florence) and by the Ethics Committees of each participating clinical center.

### Measurement of Serum Anti-Tat Antibodies

An HIV-1 B-clade Tat protein (GenBank accession no.: AAA44199.1) purchased from Diatheva (Fano, Italy) was used for anti-Tat Ab determinations. The protein was biologically active as determined by the rescue assay with HLM-1 cell line carrying a Tat-defective HIV provirus (1, 4) and/or by Tat uptake by monocyte-derived DC (MDDC) evaluated by intracellular staining for Tat in flow cytometry (36), a potency test that is used to release the Tat vaccine clinical lots.

Serum IgM, IgA, and IgG against B-clade Tat were assessed by enzyme-linked immunosorbent assay (ELISA), as previously described (35, 37). Briefly, 96-well microplates (Nunc-Immuno Plate MaxiSorp Surface; Nunc) were coated with Tat (100 ng/well) in 200 μL of 0.05 mol/L carbonate-buffer (pH 9.6), and incubated overnight at 4°C. Wells were washed 5 times with phosphate-buffered saline (PBS), pH 7.4, containing 0.05% Tween-20, by an automatic plate washer (Asys Hitech flexi wash). Wells were then saturated with PBS containing 1% bovine serum albumin (BSA) and 0.05% Tween-20 (Sigma) (blocking buffer) for 90 min at 37°C and then washed again as above. One hundred μL of patient serum samples diluted in blocking buffer at 1:100 for anti-Tat IgG detection or at 1:25 for anti-Tat IgM or IgA detection, were added to the wells and incubated at 37°C for 90 min. To correct for binding background, each sample was assessed in duplicate on Tat-coated wells and singly on the wells coated with Tat resuspension buffer. After washing, wells were saturated again with blocking buffer for 15 min at 37°C, washed again and then a goat anti-human IgG, IgM, or IgA horseradish peroxidase-conjugated secondary Ab (100 μL/well) (PIERCE-Thermo Scientific) was added to each well, and incubated for an additional 90 min at 37°C. Antigen-bound Abs were revealed by the addition of ABTS (2,2′-azino-bis(3-ethylbenzothiazoline-6-sulphonic acid) solution (Roche Diagnostics) for 60 min at 37°C. Absorbance was measured at 405 nm using a microplate reader (BIO-TEK Instruments EL800). The assay was considered valid only when both the positive and negative controls were within ±10% of variation of the absorbance values recorded in previous 50 assays. For the cut-off calculation, both the optical density (OD) readings at 405 nm of the wells coated with Tat and the delta (Δ) value were utilized. The Δ value was obtained by subtracting the OD reading of the well coated with the buffer alone from the arithmetic mean of the OD values of the two wells coated with the Tat protein. Serum samples were considered positive when the sample OD at 405 nm and Δ values were ≥0.350 and ≥0.150, respectively, as previously described (37). Ab titers ≥25 for IgM and IgA, or ≥100 for IgG were considered positive.

### Lymphocyte Phenotyping

Peripheral blood CD4^+^ and CD8^+^ T-cell counts were determined at the clinical sites by flow cytometry, using common Standard Operating Procedures (SOPs) according to standard national laboratory measurements.

### Quantification of HIV-1 DNA by Real-Time PCR

HIV-1 DNA quantification was performed by SYBR green real-time polymerase chain reaction (qPCR), as described (33, 38). DNA was obtained from 1.6 mL of peripheral blood. After incubation for 45 min at 37°C in a lysis buffer (sodium dodecyl sulfate 5%, 8 M Urea, 0.3 M NaCl, 10 mM Tris–HCl pH 7.5, 10 mM EDTA pH 8.0), DNA was purified by phenol extraction followed by ethanol precipitation and RNase treatment, and amount and quality evaluated with a NanoDrop ND-1000 Spectrophotometer by assessing the ratio of absorbance at 260 nm and 280 nm (NanoDrop Technologies, Wilmington, DE, US). Real-time PCR was performed using the HIV-1 DNA quantitative PCR kit (Diatheva s.r.l., Fano, Italy), on a 7500 Real-Time PCR System (Applied Biosystems, Foster City, CA, US). Each sample was analyzed in triplicate. Forward and reverse primers (5′-TAGCAGTGGCGCCCGA-3′ and 5′-TCTCTCTCCTTCTAGCCTCCGC-3′, respectively) were designed to amplify a 161 bp fragment derived from the 5′ long terminal repeat (LTR) U5 end to Gag-Pol start sequence of HIV-1 (GenBank accession no. AF003888). Primer specificity for HIV-1 group M was confirmed both *in silico* using BLAST (https://www.hiv.lanl.gov/content/sequence/HIV/COMPENDIUM/2012compendium.html), and by real-time PCR with different HIV-1 subtypes (B, C, F, CRF 01_AE, and CRF 02_AG), the reference strains A, D, H, and the complete DNA sequence of HIV-2 ROD (EU Programme EVA Centralized Facility for AIDS Reagents, NIBSC, UK) (38). Cross-reactivity with endogenous retroviral sequences was excluded by testing 150 HIV-1 negative blood donors (38). HIV-1 DNA copy number was estimated as described (38) using a standard curve comprising a 10-fold serial dilutions (10^5^ to 10^1^) and 2 copies dilutions of a plasmid containing the 161 bp HIV target region, including the Primer Binding Sites (PBS plasmid). The standard curve was considered valid when the slope was between −3.50 and −3.32 (93–100% efficiency) and the minimum value of the coefficient of correlation (R^2^) was 0.98. The limit of quantification was 2 copies per μg of DNA, with a detection limit of 1 copy and a dynamic range of quantification of 5 orders of magnitude (10^5^ to 10^1^). The reproducibility, assessed by calculating the mean coefficient of variation (CV%) for the threshold cycle (Ct) values, was determined as 1.4%, confirming quantification in the dynamic range. Results were expressed as log_10_ copies/10^6^ CD4^+^ T cells, calculated as the ratio between copies/μg DNA and the CD4^+^ T-cell number present in 1.5 × 10^5^ white blood cells (WBC) using the following formula: [(copies/μg DNA)/(CD4^+^/WBC) × 150,000 WBC] × 10^6^ (33).

### Quantification of HIV-1 RNA

The HIV-1 viral load (VL) in the plasma of HIV-1-infected patients was quantitatively determined using a standardized RT-PCR (AmpliPrep/COBAS® TaqMan® HIV-1 Test, version 2.0; Roche Diagnostics) that gives a linear response from 20 to 10,000,000 HIV-1 RNA copies/mL. According to manufacturer's instructions Ct values above the quantitation limit or absence of Ct were both categorized as “undetectable VL.”

The lot-specific calibration constants provided with the COBAS® AmpliPrep/COBAS® TaqMan® HIV-1 Test were used, with the Amplilink software, to calculate the titer value for the specimens and controls below the limit of detection (≥95%) of the assay (i.e., between 1 and 20 copies/mL), based upon the HIV-1 RNA and HIV-1 Quantitation Standard (QS) RNA Ct values.

### Statistical Analyses

Descriptive statistics summarizing quantitative variables included mean, standard deviation, minimum and maximum; qualitative variables were presented as number and percentage. Kaplan-Meier method was used to assess the cumulative probability of anti-Tat Ab persistence in responding participants, by vaccine regimen, and compared by the Log-Rank test. Subjects who developed anti-Tat Abs within 24 weeks of the ISS T-002 trial were defined “responders.”

Longitudinal analysis for repeated measurements (including multiple measurements per year) was used to evaluate changes from baseline of CD4^+^ and CD8^+^ T-cell number, CD4^+^/CD8^+^ T-cell ratio and HIV-1 DNA load at each year of follow up, both overall and upon stratification.

Multivariate analysis of variance for repeated measures was used in order to evaluate the effect of anti-Tat humoral responses, vaccine treatment groups, residual viremia/viral blips, CD8^+^ T-cell counts, and cART on changes from baseline of CD4^+^ T-cell counts and HIV-1 DNA load.

A longitudinal regression analysis was performed using a random-effects regression model in order to evaluate CD4^+^ T-cell number and HIV-1 DNA load over time stratified by vaccine regimen, CD4^+^ or HIV-1 DNA quartiles at baseline.

To estimate the decay rate of HIV-1 DNA, a longitudinal analysis using a random-effects regression model was performed to determine the slope of the decay from a plot of log_10_ DNA (copies/10^6^ CD4^+^ T cells) vs. time in days; the use of log plot assumes a first order kinetic decay (26).

Analysis of covariance (ANCOVA) was used to take into account the reversion to the mean (RTM) when patients were categorized for baseline CD4^+^ T-cell counts or proviral DNA quartiles (39). For this analysis, the CD4^+^ T-cell or proviral DNA measurements done at ISS T-002 baseline and at the last follow-up visit attended were used. Baseline CD4^+^ T-cell numbers or log_10_ proviral DNA copies per million CD4^+^ T cells and quartiles were the model covariates.

A generalized estimating equation with adjustment for repeated measures in the same volunteers was performed in order to evaluate relationship between CD4^+^/CD8^+^ T-cell ratio, CD4^+^ T-cell number, CD8^+^ T-cell number and HIV-1 DNA load.

The Spearman Correlation Coefficient was used in order to test the correlation between CD4^+^/CD8^+^ T-cell ratio, CD4^+^ T cells, CD8^+^ T cells, and HIV-1 DNA load.

Statistical analyses were carried out at two-sided with a 0.05 significance level, using SAS® (Version 9.4, SAS Institute Inc., Cary, NC, USA) and STATA^tm^ version 8.2 (Stata Corporation, College Station, Texas, United States).

## Results

### Study Participants

Ninety-three volunteers out of the 155 participants evaluated for the ISS T-002 trial agreed to enter the extended follow-up study (Supplementary Figure 1). One patient was excluded from all the analyses because of persistent non-compliance with cART. Five patients dropped out of the study: 1 after 24 weeks (discontinued cART), 1 after 36 weeks (relocated), 1 after 48 weeks (not compliant to study visits), 1 after 48 weeks (death due to lung tumor) and 1 after 84 weeks (lost to follow-up). Table 1 reports the baseline characteristics of the ISS T-002 EF-UP study participants and the comparison with ISS T-002 baseline. Seventy-seven out of the 92 evaluable volunteers were male (83.7%). With regard to vaccine regimen, 21 volunteers had received Tat at 7.5 μg, 3 × ; 20 Tat at 7.5 μg, 5 × ; 27 Tat at 30 μg, 3 × ; and 24 Tat at 30 μg, 5 × (Table 1). Seventy-eight percent of the patients had a CD4^+^ T-cell nadir >250 cells/μL. Volunteers entered the study at different time periods after the first immunization (median time 200 weeks) with a range comprised between week 144 and 304 (the week at study entry was taken as week 0 of the extended follow-up protocol). They were followed-up for a median of 120 weeks (range 24–132). The median time of follow-up since the first immunization was 316 weeks (range 185–412). At ISS T-002 EF-UP study entry, volunteers were in cART for a mean of 10 years.

**Table 1:** Baseline characteristics of the ISS T-002 EF-UP study participants and comparison with ISS T-002 baseline.

	**Population**	
**GENDER**		
Male	77 (83.7)	
Female	15 (16.3)	
**TREATMENT GROUP^#^, *N*. (%)**		
Tat 7.5 μg, 3X	21 (22.8)	
Tat 7.5 μg, 5X	20 (21.7)	
Tat 30 μg, 3X	27 (29.4)	
Tat 30 μg, 5X	24 (26.1)	
**CD4**^**+**^ **T-CELL NADIR**		
≤250 cells/μL	21.7%	
>250 cells/μL	78.3%	
	**ISS T-002 baseline**	**ISS T-002 EF-UP baseline**
**AGE**		
Mean ± s.d.^a^	42 ± 7	46 ± 7
Range	24–55	27–59
**CD4**^**+**^ **T-CELLS (CELLS/μL)**		
Mean ± s.d.	623 ± 208	704 ± 23
Range	144–1,490	336–1,461
**CD4**^**+**^ **T-CELLS (%)**		
Mean ± s.d.	33.6 ± 8	35.6 ± 8.6
Range	14–60.7	14.9–62.2
**CD8**^**+**^ **T-CELLS (CELLS/μL)**		
Mean ± s.d.	774 ± 400	747 ± 343
Range	143–2915	199–2,068
**CD8**^**+**^ **T-CELLS (%)**		
Mean ± s.d.	38.4 ± 10.1	35.9 ± 9.5
Range	18.1–63.7	17–60
**CD4**^**+**^**/CD8**^**+**^ **T-CELL RATIO (%)**		
Mean ± s.d.	1.0 ± 0.5	1.1 ± 0.5
Range	0.2–2.3	0.3–2.8
**HIV RNA**		
<40 copies/mL) – N. (%)	87 (94.6)	85 (93.4)
>40 copies/mL – N. (%)	5 (5.4)	6 (6.6)
Mean copies/mL ± s.d.	70 ± 13.1	182.2 ± 245.5
Range	57.8–90.9	50.3–678^*^
**YEARS FROM HIV DIAGNOSIS**		
Mean ± s.d.	9 ± 6	13 ± 6
Range	1–25	4–29
**YEARS FROM cART INITIATION**		
Mean ± s.d.	6 ± 5	10 ± 5
Range	0.5–19	4–22
**CURRENT cART REGIMEN**		
NNRTI- or NRTI-based	64.1%	66.3%
PI-based	35.9%	30.4%
Other	0.0%	3.3%

# Tat was administered intradermally, without adjuvants, at 7.5 or 30 μg doses, given 3 × or 5 × , 1 month apart. n indicates the number of evaluable individuals;

a Standard deviation;

* *All patients were cART compliant*.

### Anti-Tat Antibodies

**Figure 1:**
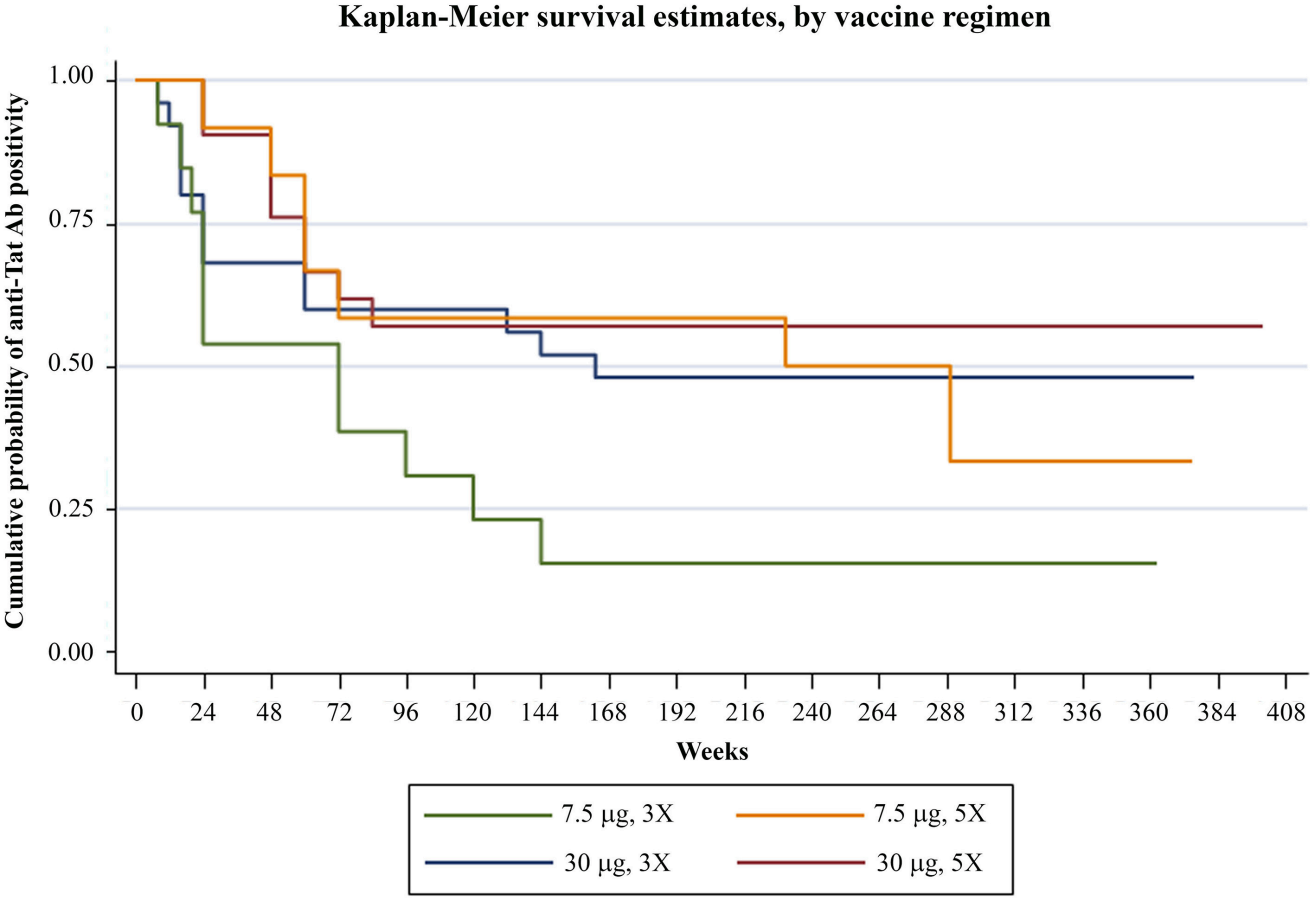
Anti-Tat Ab persistence by vaccine treatment groups. Kaplan-Meier estimates showing the cumulative probability of anti-Tat Ab durability in ISS T-002 responders (*n* = 71) stratified according to treatment groups up to 412 weeks of follow-up (median follow-up: 316 weeks).

Analysis of humoral responses indicated that anti-Tat Abs persisted for the entire period of follow-up in a high proportion of participants, particularly in the Tat 30 μg regimens (49% of the vaccinees) (Figure 1). In fact, as compared to the 7.5 μg regimens, volunteers vaccinated with the 30 μg Tat regimens showed persistent anti-Tat IgG and IgA Abs (log-rank Test *p* = 0.0179 and 0.0128, respectively, data not shown) and those who developed anti-Tat Abs of all 3 classes were more likely to maintain the Ab response to Tat (at least 1 class) [66 vs. 23% (2 classes) and 27% (1 class), Log-Rank Test *p* = 0.0007] (Supplementary Figure 2).

### CD4^+^, CD8^+^ T-Cells, and CD4^+^/CD8^+^ T-Cell Ratio

Analysis of immunological parameters of the 92 vaccinees was performed by years of follow-up, starting from the ISS T-002 study entry, that is, from year 1 to 8.

**Figure 2:**
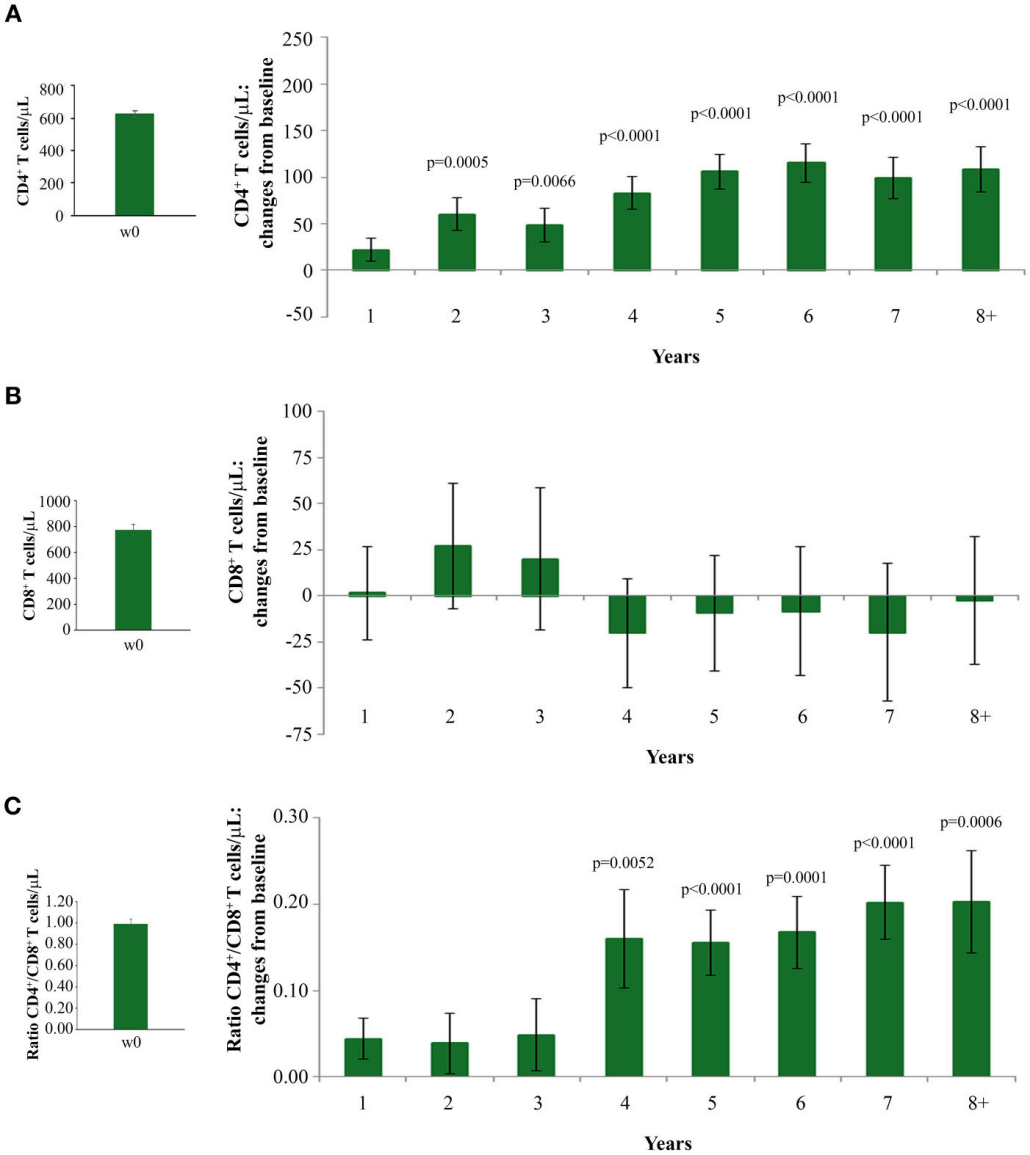
Changes over baseline of CD4^+^ T-cells **(A)** CD8^+^ T-cells **(B)** and CD4^+^/CD8^+^ T-cell ratio **(C)** during 8 years of follow-up. Baseline values (left panels) and annual changes over baseline (right panels) from ISS T-002 study entry of CD4^+^ T-cells **(A)**, CD8^+^ T-cells **(B)**, and CD4^+^/CD8^+^ T-cell ratio **(C)** in all vaccinees are shown. The number of participants tested are as follows. CD4^+^ T-cells: year 1 *n* = 92, year 2 *n* = 63, year 3 *n* = 43, year 4 *n* = 37, year 5 *n* = 52, year 6 *n* = 82, year 7 *n* = 58 year 8+ *n* = 37; CD8^+^ T-cells and CD4^+^/CD8^+^ T-cell ratio: year 1 *n* = 91, year 2 *n* = 59, year 3 *n* = 41, year 4 *n* = 35, year 5 *n* = 48, year 6 *n* = 78, year 7 *n* = 54, year 8+ *n* = 34. Data are presented as mean values with standard error. A longitudinal analysis for repeated measurements was applied. *p*-values assess the values at year 1–8 after immunization vs. baseline values.

The statistically significant increase of CD4^+^ T-cell counts recorded since year 2 continued over time, reaching levels higher than 100 cells/μL over baseline values since year 5, gains which were maintained through year 8 (Figure 2A).

Increases of CD4^+^ T-cells were similar with non-nucleoside reverse transcriptase (NNRTI)/nucleoside reverse transcriptase (NRTI)- or PI-based treatments (data not shown).

Interestingly, subjects with CD4^+^ T-cell nadir ≤250 cells/μL had also statistically significant CD4^+^ T-cell increases from baseline, which were equal or even greater than those observed in vaccinees with a higher nadir (Supplementary Figure 3). This is of clinical relevance, since subjects with a nadir ≤250 cells/μL are known to respond insufficiently to cART (40, 41) or vaccination (42).

CD8^+^ T-cell levels remained stable throughout the 8 years of follow-up (Figure 2B). The increase of CD4^+^ T-cell counts and the maintenance of CD8^+^ T-cell number after vaccination resulted in a statistically significant increase of the CD4^+^/CD8^+^ T-cell ratio by year 4, which persisted through year 8 of follow-up (Figure 2C).

**Figure 3:**
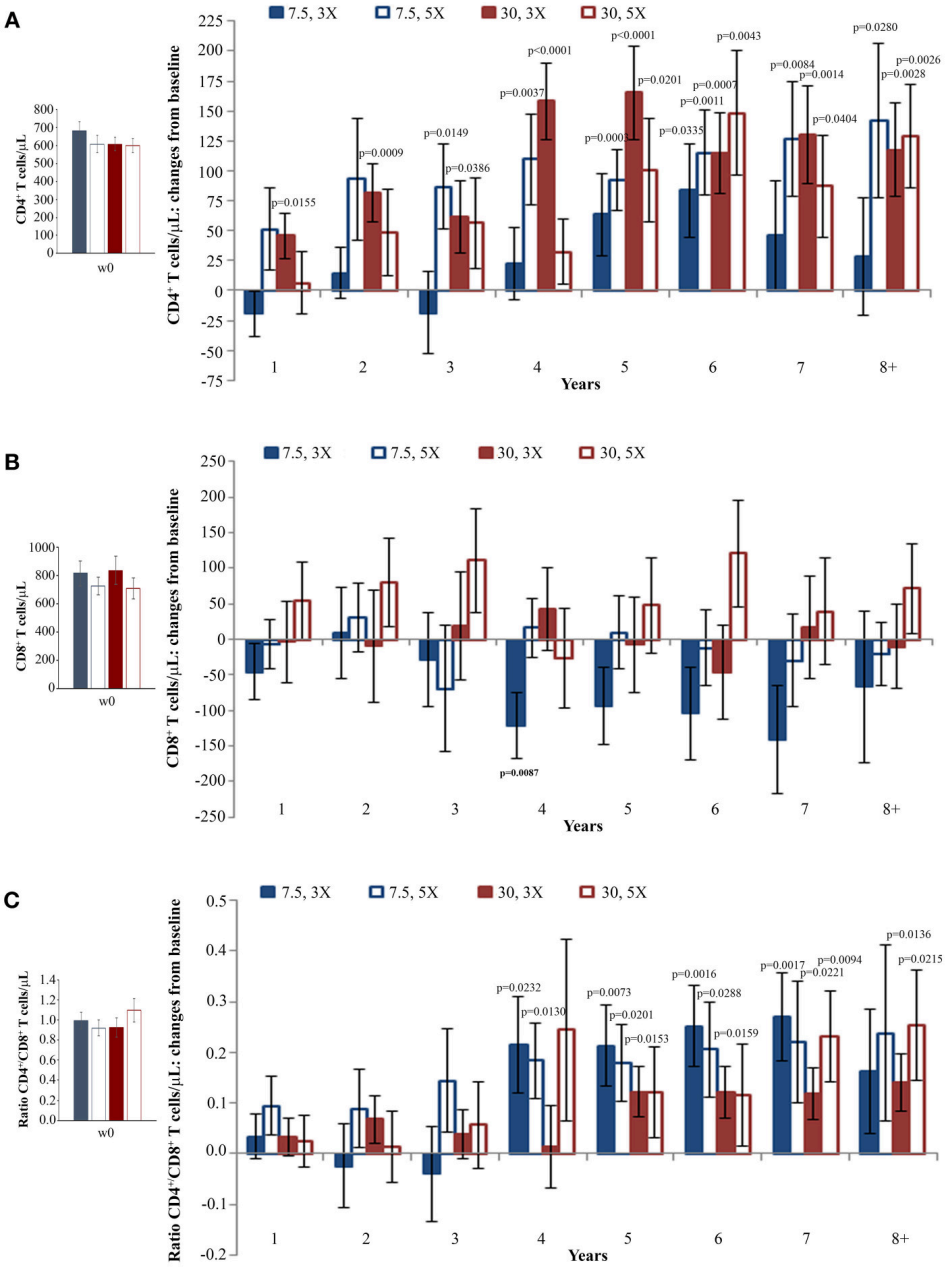
CD4^+^ T-cell and CD8^+^ T-cell numbers, and CD4^+^/CD8^+^ T-cell ratio during follow-up stratified by Tat vaccine regimens. Baseline values (left panels) and annual changes (right panels) from ISS T-002 trial baseline of CD4^+^ T-cells **(A)**, CD8^+^ T-cells **(B)**, and CD4^+^/CD8^+^ T-cell ratio **(C)** over 8 years of follow-up are shown according to the Tat vaccine regimen. The number of participants tested are as follows: Tat 7.5 μg, 3 ×: year 1 *n* = 21, year 2 *n* = 14, year 3 *n* = 9, year 4 *n* = 10, year 5 *n* = 13, year 6 *n* = 18, year 7 *n* = 12 year 8+ *n* = 7; Tat 7.5 μg, 5 ×: year 1 *n* = 20, year 2 *n* = 14, year 3 *n* = 7, year 4 *n* = 10, year 5 *n* = 13, year 6 *n* = 19, year 7 *n* = 12 year 8+ *n* = 7; Tat 30 μg, 3 ×: year 1 *n* = 27, year 2 *n* = 17, year 3 *n* = 14, year 4 *n* = 10, year 5 *n* = 15, year 6 *n* = 25, year 7 *n* = 19 year 8+ *n* = 11; Tat 30 μg, 5 ×: year 1 *n* = 24, year 2 *n* = 18, year 3 *n* = 134, year 4 *n* = 7, year 5 *n* = 13, year 6 *n* = 20, year 7 *n* = 15 year 8+ *n* = 12. Data are presented as mean values with standard error. A longitudinal analysis for repeated measurements was applied. *p*-values assess the values at year 1–8 after immunization vs. baseline values.

When the immunization regimen was considered, the greatest effects of Tat vaccination were observed with the Tat 30 μg, 3 ×, with increases of CD4^+^ T cells up to 165 cells/μL (Figure 3A), substantially stable levels of CD8^+^ T cells (Figure 3B), and significant increases of the CD4^+^/CD8^+^ T-cell ratio over time (Figure 3C).

In order to better evaluate Tat vaccine dose-effects on the increase of CD4^+^ T cells, a longitudinal regression analysis using a random-effect regression model was used for the four vaccine regimens. This analysis showed statistically significant increases of CD4^+^ T cells for all regimens with the highest increase in the two Tat 30 μg regimens, while CD4^+^ T-cell gains in the two Tat 7.5 μg regimens were more modest (Supplementary Figure 4 and Supplementary Data Sheet 1).

### HIV Proviral DNA in Blood

**Figure 4:**
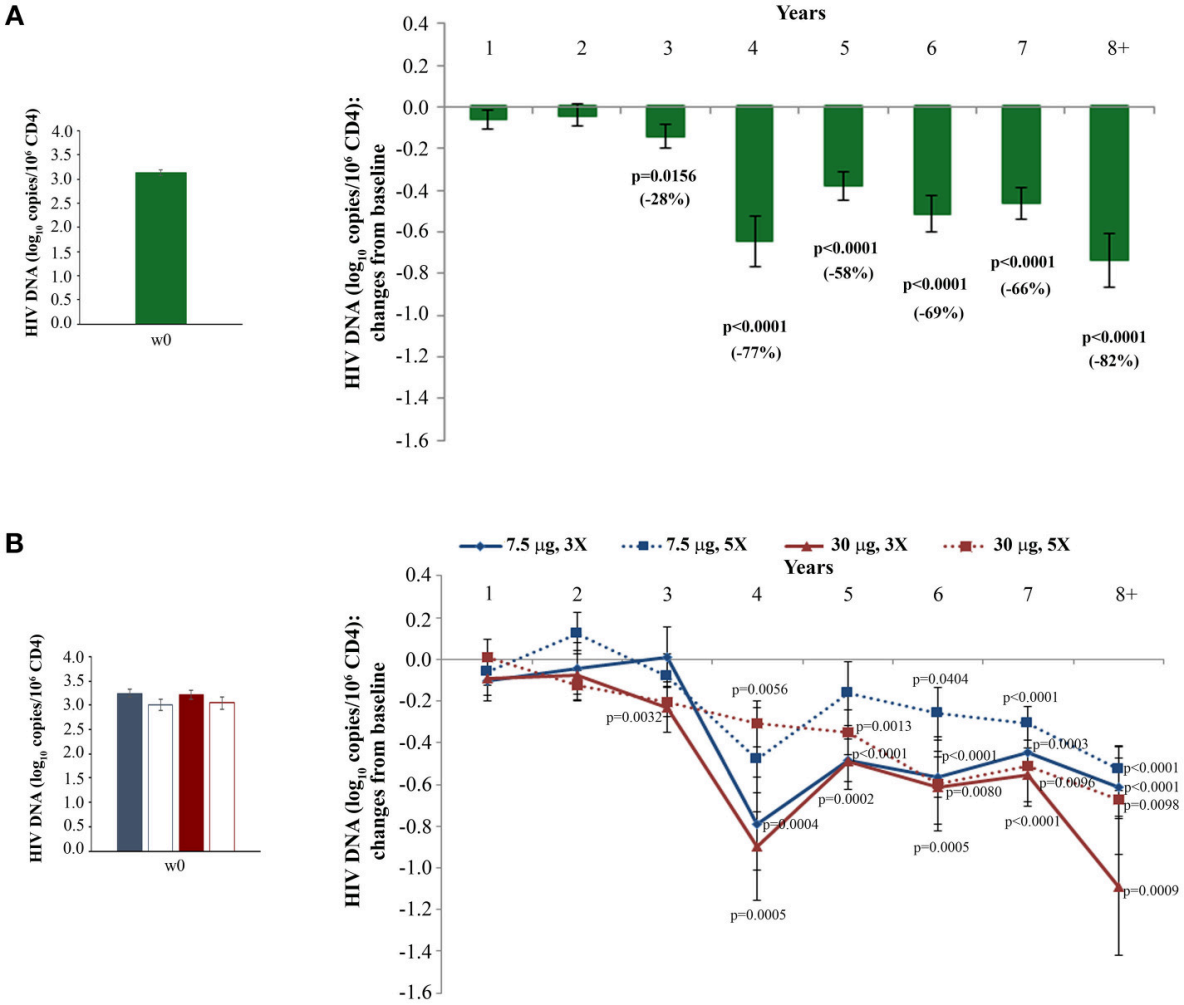
Changes of blood HIV DNA load over baseline in all vaccinees **(A)** and stratified by treatment groups **(B)** during follow-up. Baseline values (left panels) and annual changes (right panels) of HIV DNA levels (expressed as log_10_ copies/10^6^ CD4^+^ T-cells) from ISS T-002 study entry in all vaccinees **(A)**, and stratified by Tat vaccine treatment groups **(B)** are shown. The number of participants tested are as follows: year 1 *n* = 89, year 2 *n* = 59, year 3 *n* = 42, year 4 *n* = 36, year 5 *n* = 51, year 6 *n* = 75, year 7 *n* = 58, year 8+ *n* = 37. Data are presented as mean values with standard error. A longitudinal analysis for repeated measurements was applied. *p*-values assess the values at year 1–8 after immunization vs. baseline values.

HIV proviral DNA in peripheral CD4^+^ T cells was evaluated longitudinally over the whole follow-up period. As shown in Figure 4A, HIV-1 DNA levels continued to decrease over time with changes from baseline becoming statistical significant from year 3, reaching a −82% mean reduction (−0.72 log_10_ copies/10^6^ CD4^+^ T cells) at year 8 as compared to study entry. Although all vaccine regimens had very similar baseline HIV-1 DNA mean values, the Tat 30 μg, 3 × regimen was the most effective at reducing proviral DNA load, reaching at year 8 a decrease of −92% (−1.1 log_10_ copies/10^6^ CD4^+^ T cells), followed by the Tat 30 μg, 5 × that showed a decrease of −79% (−0.7 log_10_ copies/10^6^ CD4^+^ T cells), Tat 7.5 μg, 3 ×, which had very similar kinetics of proviral decay reaching at year 8 a decrease of −76% (−0.6 log_10_ copies/10^6^ CD4^+^ T cells), while Tat 7.5 μg, 5 × was the least effective with a proviral DNA reduction of −70% (−0.5 log_10_ copies/10^6^ CD4^+^ T cells, at year 8) (Figure 4B).

Moreover, although in the first 3 years of follow-up a significant reduction of HIV-1 DNA was seen only in vaccinees under PI-based regimens (33), since year 4 of follow-up both NNRTI/NRTI- and PI-based cART regimens were associated with similar, persistent and statistically significant reductions of proviral DNA (Supplementary Figure 5).

These results were confirmed in a longitudinal regression analysis using the random-effects regression model after stratification of the results according to the vaccine regimen. A marked reduction of HIV proviral DNA was again observed in all vaccine regimens, with the greatest decrease in the Tat 30 μg, 3 ×, followed by Tat 7.5 μg, 3 × and Tat 30 μg, 5 ×, while Tat 7.5 μg, 5 × showed the smallest HIV proviral DNA decay (Supplementary Figure 6 and Supplementary Data Sheet 1).

**Figure 5:**
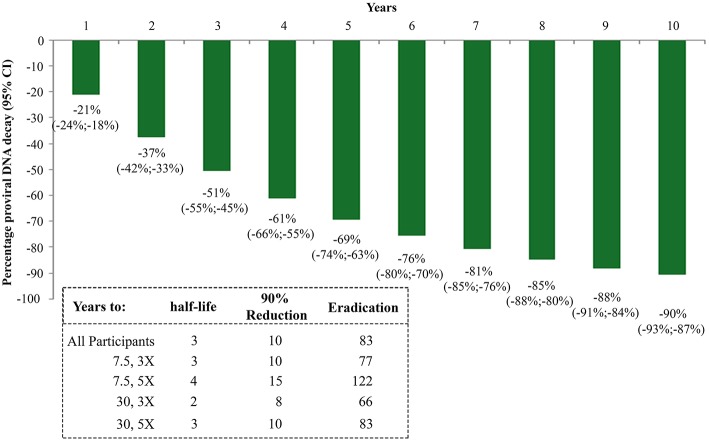
Kinetics parameter estimates of proviral DNA decay in vaccinees stratified by Tat vaccine regimens. Estimates of HIV-1 DNA annual decay in all vaccinees expressed as the percentage of HIV-1 DNA decay with 95% confidence interval (upper panel) or years to 50% reduction [half-life (t_1/2_)], to 90% reduction and to eradication for all vaccinees and by Tat vaccine regimens (lower panel) are shown.

The random-effects regression model was then used to estimate the rate of HIV-1 DNA decay. As shown in Figure 5, a HIV-1 DNA half-life (t_1/2_) of 3 years and a 90% reduction of the DNA reservoir in 10 years were estimated for the whole study population. However, these estimates varied markedly according to the vaccine regimen. In fact, t_1/2_ and time to 90% reduction were shortest for the Tat 30 μg, 3 × group (t_1/2_ = 2 years; 90% reduction = 8 years), followed by Tat 7.5 μg, 3 × (t_1/2_ = 3 years; 90% reduction = 10 years), Tat 30 μg, 5 × (t_1/2_ = 3 years; 90% reduction = 10 years), and Tat 7.5 μg, 5 × (t_1/2_ = 4 years; 90% reduction = 15 years) (Figure 5).

The time required for proviral DNA eradication was calculated assuming the average frequency of lymphocytes harboring proviral DNA to be about 300/10^6^ (43) in a background of 10^12^ total body lymphocytes (44). Accordingly, the eradication times were estimated to be 83 years for the whole population, and 66, 77, 83, 122 years for the 30 μg, 3 ×; 7.5 μg, 3 ×; 30 μg, 5 × and 7.5 μg, 5 × group, respectively (Figure 5).

### Transient Detectable Viremia and Blood Proviral DNA Decay

**Figure 6:**
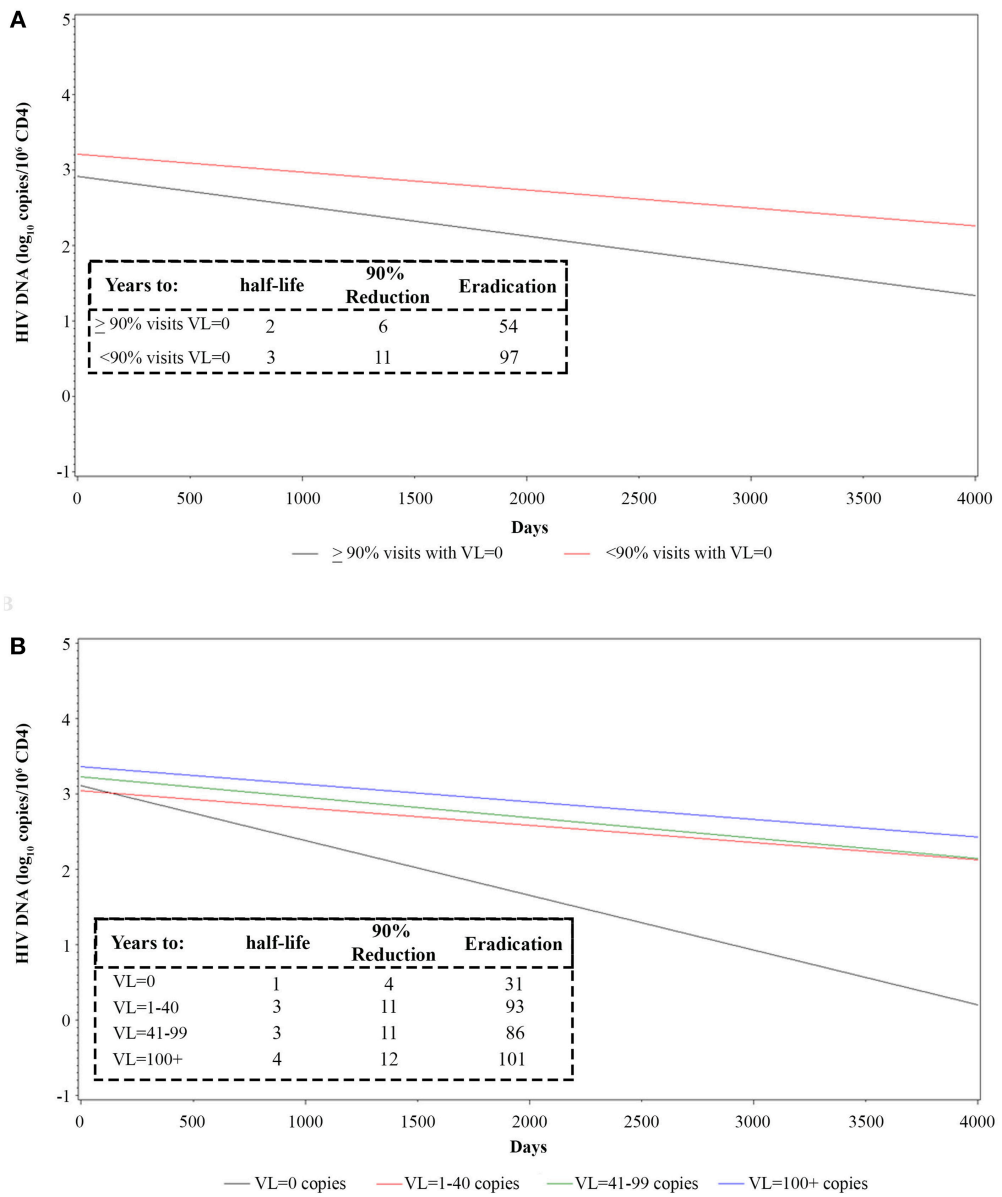
Longitudinal regression analysis and estimates of proviral HIV DNA decay in vaccinees stratified by frequency of intermittent viremia **(A)** or viremic classes **(B)**. Effect of transient viremic events on proviral DNA decay (log_10_ copies/10^6^ CD4^+^ T-cells) in vaccinees stratified by the percentage of visits with detectable VL measurements (VL = 0 copies/mL in ≥90 or <90% visits) **(A)** is shown. Effect of transient viremic events on proviral DNA decay in vaccinees stratified by VL intensity classes: persistent aviremic individuals (0 HIV RNA copies/mL) vs. subjects with 1–40 HIV RNA copies/mL, 41–99 HIV RNA copies/mL or ≥100 HIV RNA copies/mL **(B)** are shown. Insets: DNA decay kinetics parameter estimates for VL frequency **(A)** or intensity classes **(B)** are shown.

The transient presence of detectable plasma VL in otherwise virologically suppressed individuals on ART appears to be associated with a slower decay of HIV proviral DNA (45–47). We therefore examined the relationship between the frequency and the level of intermittent residual viremia/viral blips and proviral DNA decay. To this purpose, regression analyses were made in vaccinees grouped by either the frequency of visits with complete virological suppression (<90 or ≥90% of visits with VL = 0) or by VL cut-off intervals (0; 1–40; 41–99; ≥100 RNA copies/mL) (see also Supplementary Table 1). A faster HIV DNA decay with a shorter proviral DNA half-life, time to 90% reduction and time to eradication, was observed in vaccinees with VL = 0 in ≥90% of visits as compared to <90% of visits (Figure 6A and Supplementary Data Sheet 1). When vaccinees were stratified according to VL cut-off classes, the fastest HIV DNA decay was observed in patients in the lowest VL class (VL = 0), whereas the slowest kinetics were observed in vaccinees experiencing blips above 100 RNA copies/mL; intermediate kinetics of DNA decay were observed in vaccinees with intermediate viremic measurements (Figure 6B and Supplementary Data Sheet 1). These data suggested replenishment of the HIV-1 proviral DNA reservoir by residual viremia and/or viral blips.

### CD4^+^ T-Cells and Proviral DNA After Stratification by Quartiles of CD4^+^ or Proviral DNA at Study Entry

To evaluate whether the individual immunological status or size of latent HIV reservoir of the volunteers might have affected the response to Tat vaccination, the kinetics of CD4^+^ T-cell increases or proviral DNA decays were evaluated upon stratification by quartiles according to CD4^+^ T-cell number or proviral DNA at ISS T-002 study entry. The data were then analyzed by three models including (i) changes from baseline upon time by a longitudinal analysis for repeated measurements, (ii) variations of absolute CD4^+^ T-cell numbers or proviral DNA copies upon time by a random-effects linear regression model, and (iii) single paired pre-post vaccination measures by ANCOVA, to take into account the RTM effect.

**Figure 7:**
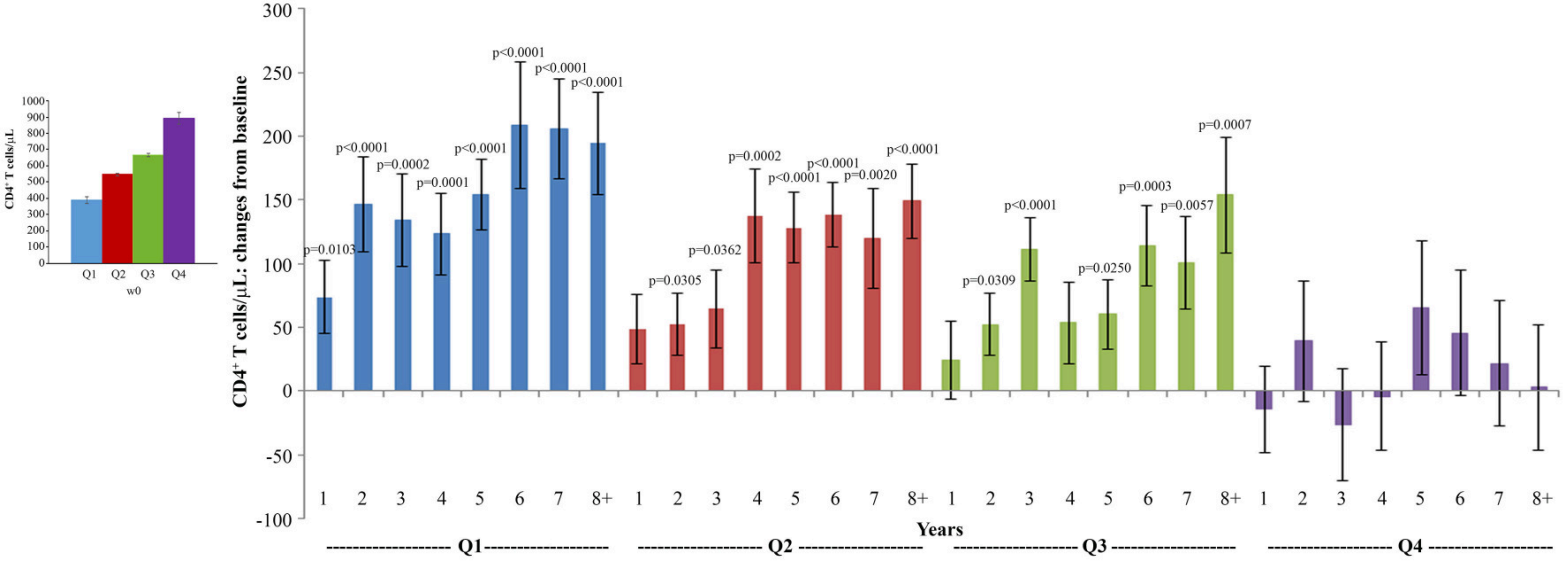
Analysis of variations of CD4^+^ T-cells stratified according to baseline quartiles by longitudinal analysis for repeated measurements. Longitudinal analysis for repeated measurements of changes of CD4^+^ T-cell number (cells/μL) from baseline is shown. Left panel: baseline quartiles values; right panel: annual changes by baseline quartiles. Data (*n* = 91) are presented as mean values with standard error. CD4^+^ T-cell quartiles at baseline: Q1 <493 (*n* = 23), Q2 493–600 (*n* = 24), Q3 601–734 (*n* = 22) and Q4 >734 (*n* = 22). The number of participants tested are as follows: year 1 *n* = 89, year 2 *n* = 59, year 3 *n* = 42, year 4 *n* = 36, year 5 *n* = 51, year 6 *n* = 75, year 7 *n* = 58, year 8+ *n* = 37. *p*-values assess the values at year 1–8 after immunization vs. baseline values. The analysis includes multiple measurements for each year.

The analysis of CD4^+^ T-cell changes from baseline over time showed that subjects with the lowest CD4^+^ T-cell counts at baseline (Q1: CD4^+^ T cells <493/μL) experienced very early (year 1) and statistically significant increases of CD4^+^ T cells, followed by those in Q2 (CD4^+^ T cells 493–600/μL) and in Q3 (CD4^+^ T cells: 601–734/μL) in whom statistically significant increases of CD4^+^ T cells were recorded since year 2 of follow-up, while the increase observed in Q4 (CD4^+^ T cells >734/μL) were not statistically significant (Figure 7).

The analysis of absolute CD4^+^ T-cell kinetics by longitudinal regression analysis showed slightly different results, with significant increases of CD4^+^ T cells for all quartiles, including Q4, although the greatest increases were observed in Q1, followed by Q2 and Q3 as compared to Q4 (Supplementary Figure 7 and Supplementary Data Sheet 1). In particular, Q1 showed the highest CD4^+^ T-cell increase, with the 95% CI of the Q1 regression curve transitioning into the 95% CI of the Q2 curve, suggesting positive effects of Tat vaccination in poor immunological responders.

When the RTM effect was taken into account by ANCOVA, the post-vaccination means of CD4^+^ T cells were not found to be significantly different among quartiles (data not shown), indicating that Tat vaccination has similar effects irrespective of the CD4^+^ T-cell number at study entry.

**Figure 8:**
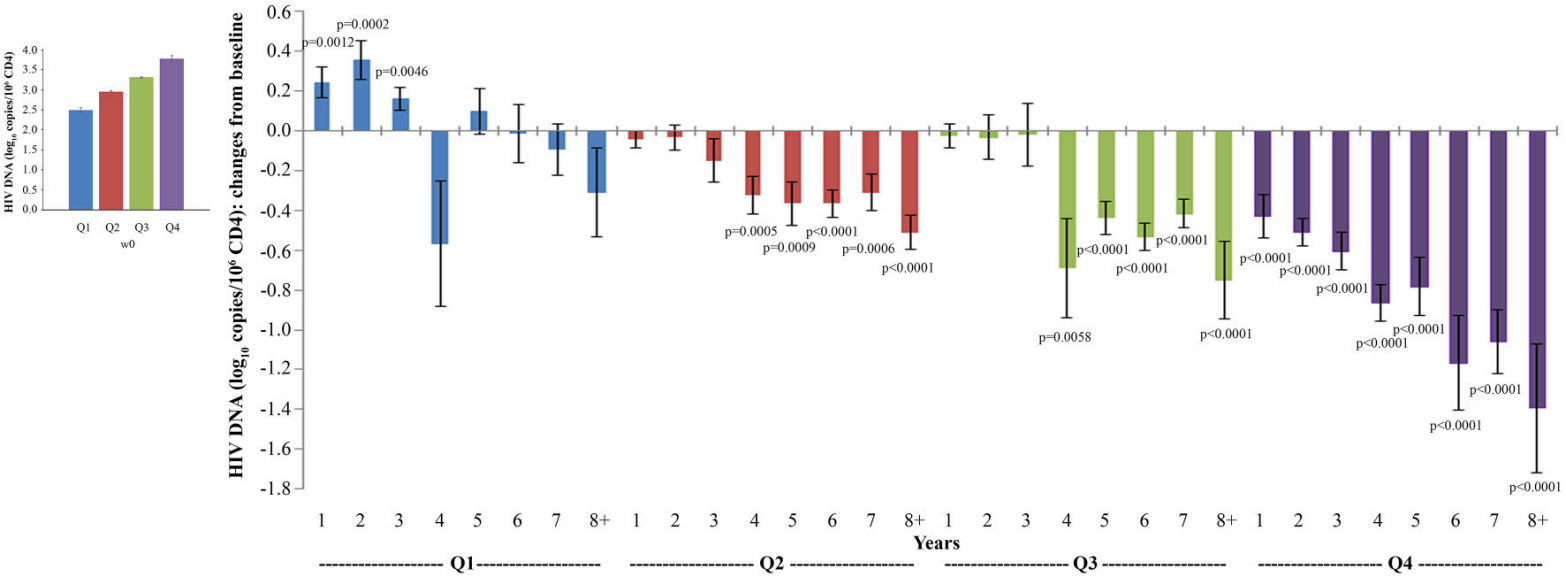
Analysis of variations of HIV-1 proviral DNA stratified according to baseline quartiles by longitudinal analysis for repeated measurements. Longitudinal analysis for repeated measurements of changes of HIV-1 proviral DNA (log_10_ copies/10^6^ CD4^+^ T-cells) from baseline is shown. Left panel: baseline quartiles values; right panel: annual changes by baseline quartiles. Data (*n* = 91) are presented as mean values with standard error. HIV-1 proviral DNA quartiles at baseline: Q1 <2.86 (*n* = 22), Q2 2.86–3.10 (*n* = 24), Q3 3.11–3.47 (*n* = 23) and Q4 >3.47 (*n* = 22). The number of participants tested are as follows: year 1 *n* = 89, year 2 *n* = 59, year 3 *n* = 42, year 4 *n* = 36, year 5 *n* = 51, year 6 *n* = 75, year 7 *n* = 58, year 8+ *n* = 37. *p*-values assess the values at year 1–8 after immunization vs. baseline values. The analysis includes multiple measurements for each year.

Similar but inverse relationships among quartiles were observed for proviral DNA decay. When proviral DNA kinetics were analyzed as changes from baseline over time, vaccinees with the highest proviral DNA load at study entry (Q4) experienced the earliest (year 1), progressive and most pronounced DNA load reduction (from −0.4 to −1.3 HIV-1 DNA log_10_ copies/10^6^ CD4^+^ T-cells), followed by vaccinees in Q3 and Q2, in whom DNA reduction was less pronounced and became significant only since year 4; significant proviral DNA load increases were observed in Q1 from year 1 to 3, while reductions thereafter were not significant (Figure 8).

Conversely, when absolute proviral DNA copies stratified by baseline quartiles were analyzed by the random-effects regression model, a significant decay was observed in all quartiles including Q1, with the greatest decay for Q4 followed by Q3, while Q2 and Q1 showed the lowest proviral decay (Supplementary Figure 8 and Supplementary Data Sheet 1).

Finally, when the RTM effect was taken into account by ANCOVA, the post-vaccination means of proviral DNA (log_10_) copies were not found to be significantly different among quartiles (data not shown), indicating similar effects of vaccination irrespective of the size of the reservoir at study entry.

### Correlation of the CD4^+^/CD8^+^ T-Cell Ratio, CD4^+^ or CD8^+^ T-Cell Number With the HIV Proviral DNA Size

**Figure 9:**
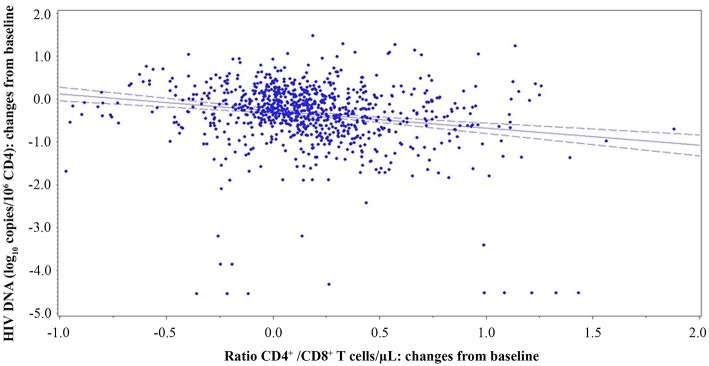
Relationship of CD4^+^/CD8^+^ T-cell ratio and proviral DNA in vaccinees during follow-up. Relationships between changes of HIV proviral DNA levels from baseline (log_10_ copies/10^6^ CD4^+^ T-cells) and the changes of CD4^+^/CD8^+^ T-cell ratio from baseline are shown. A generalized estimating equation with adjustment for repeated measures was utilized.

The CD4^+^/CD8^+^ T-cell ratio has been indicated to correlate inversely with the HIV reservoir size (48, 49). Therefore, analyses were made to verify the presence of a correlation between proviral DNA decay and the CD4^+^/CD8^+^ T-cell ratio, CD4^+^ or CD8^+^ T-cell counts. A significant inverse relationship was observed between the variations over time of HIV proviral DNA and the CD4^+^/CD8^+^ T-cell ratio (both measured as changes from baseline; β = −0.3385; *p* = 0.0246) using a generalized estimating equation (GEE) with adjustment for repeated measures (Figure 9).

An inverse correlation was also observed with CD4^+^ T-cell changes from baseline (Spearmen correlation coefficient = −0.11034; *p* = 0.0005), although the GEE analysis did not reach statistical significance (Supplementary Figure 9A).

In contrast, a significant direct relationship between HIV proviral DNA and CD8^+^ T-cell changes from baseline (β = 0.0002; *p* = 0.0166) was revealed by the GEE analysis (Supplementary Figure 9B).

### Multivariate Analysis of CD4^+^ T-Cells and Proviral DNA vs. Humoral Anti-Tat Responses, Vaccine Treatment Groups, Transient Viremic Events, CD8^+^ T-Cells, and Antiretroviral Therapy

We next investigated the effect of anti-Tat humoral responses, vaccine treatment regimens, residual viremia/viral blips, cART, and CD8^+^ T-cells on the kinetics of CD4^+^ T-cell counts or HIV-1 DNA decay by a multivariate regression model, in which the effect of these variables were evaluated on the changes from baseline of CD4^+^ T-cell counts and HIV-1 DNA copy number (Tables 2, 3). For this analysis, VL was categorized in two groups according to the frequency of visits with undetectable viremia (i.e., ≥90 or <90% with VL = 0, see Figure 6A).

**Table 2:** CD4^+^ T-cells vs. anti-Tat humoral immune response, vaccine treatment groups, viral load, and antiretroviral drug regimens.

**CD4^**+**^ T-cells**	**Year^a^**	**Estimate^b^**	**95% CI^c^**		***p*-Value**
**ANTI-TAT HUMORAL RESPONSE**					
IgM^+^, IgG^+^, and IgA^+^	1	32	−32	96	0.3246
	2	101	10	192	0.0296
	3	89	−19	197	0.1078
	4	117	0	233	0.0492
	5	194	79	309	0.0009
	6	145	34	257	0.0106
	7	79	−26	183	0.1420
	8+	75	−20	169	0.1221
IgM^+^/IgG^+^ or IgG^+^/IgA^+^ or IgM^+^/IgA^+^	1	50	−15	115	0.1293
	2	53	−21	127	0.1575
	3	30	−28	88	0.3071
	4	175	42	309	0.0099
	5	179	44	313	0.0092
	6	140	41	239	0.0058
	7	95	−1	192	0.0528
	8+	69	−20	158	0.1277
IgM^+^ or IgG^+^ or IgA^+^	1	13	−34	61	0.5818
	2	55	−7	117	0.0815
	3	2	−126	130	0.9771
	4	63	−4	131	0.0661
	5	162	79	246	0.0001
	6	88	12	164	0.0241
	7	65	−62	193	0.3148
	8+	−57	−198	85	0.4311
Ab Negative	1	55	−7	117	0.0834
	2	72	−32	175	0.1745
	3	38	−35	110	0.3090
	4	118	−24	261	0.1039
	5	116	33	199	0.0062
	6	137	38	235	0.0064
	7	132	24	239	0.0162
	8+	211	91	332	0.0006
**VACCINE TREATMENT GROUPS**					
Tat 7.5 μg, 3 ×	1	−7	−53	39	0.7597
	2	22	−33	76	0.4383
	3	10	−89	109	0.8417
	4	36	−79	152	0.5352
	5	115	15	216	0.0246
	6	93	−3	189	0.0565
	7	59	−43	161	0.2558
	8+	68	−26	162	0.1557
Tat 7.5 μg, 5 ×	1	67	−18	152	0.1240
	2	129	−12	270	0.0738
	3	46	−46	138	0.3293
	4	135	33	237	0.0096
	5	149	47	251	0.0044
	6	124	24	224	0.0154
	7	91	−30	212	0.1410
	8+	64	−54	182	0.2904
Tat 30 μg, 3 ×	1	65	17	112	0.0080
	2	69	10	128	0.0227
	3	27	−44	98	0.4547
	4	197	80	314	0.0009
	5	222	117	327	<0.0001
	6	130	42	218	0.0039
	7	128	28	228	0.0122
	8+	78	−12	168	0.0905
Tat 30 μg, 5 ×	1	26	−31	84	0.3712
	2	62	−10	135	0.0932
	3	75	−5	156	0.0663
	4	105	−16	226	0.0877
	5	165	60	270	0.0020
	6	162	54	271	0.0032
	7	93	−7	194	0.0684
	8+	89	−16	194	0.0983
**VIRAL LOAD**					
At least 90% visits with 0 copies	1	60	−22	142	0.1517
	2	84	−5	174	0.0653
	3	46	−47	139	0.3331
	4	163	12	313	0.0343
	5	234	93	375	0.0011
	6	134	25	243	0.0162
	7	92	−5	189	0.0632
	8+	63	−21	148	0.1389
Less than 90% visits with 0 copies	1	15	−9	39	0.2142
	2	56	8	105	0.0215
	3	33	−22	89	0.2372
	4	74	32	117	0.0006
	5	92	53	131	<0.0001
	6	121	70	171	<0.0001
	7	94	30	157	0.0037
	8+	86	15	156	0.0172
**ARV REGIMENS**					
NNRTI- or NRTI-based		84	41	126	0.0001
PI-based		97	33	161	0.0029

a Year since first immunization;

b Least square means;

c *Confidence interval*.

*Multivariate analysis of variance for repeated measures of longitudinal samples from 92 individuals*.

**Table 3:** HIV-1 DNA vs. anti-Tat humoral immune response, vaccine treatment groups, viral load, CD8^+^T-cell counts from baseline and antiretroviral drug regimens.

**HIV-1 DNA (log10 copies/10^**6**^ CD4^**+**^)**	**Year^a^**	**Estimate^b^**	**95% CI^c^**		***p*-Value**
**ANTI-TAT HUMORAL RESPONSE**					
IgM^+^, IgG^+^, and IgA^+^	1	−0.306	−0.485	−0.127	0.0008
	2	−0.049	−0.287	0.190	0.6892
	3	−0.307	−0.624	0.011	0.0581
	4	−1.185	−1.563	−0.806	<0.0001
	5	−0.461	−0.731	−0.190	0.0008
	6	−0.768	−1.231	−0.305	0.0012
	7	−0.710	−0.963	−0.458	<0.0001
	8+	−1.312	−1.876	−0.747	<0.0001
IgM^+^/IgG^+^ or IgG^+^/IgA^+^ or IgM^+^/IgA^+^	1	−0.138	−0.450	0.175	0.3880
	2	−0.241	−0.605	0.123	0.195
	3	−0.376	−0.700	−0.051	0.0234
	4	−0.654	−1.157	−0.150	0.0109
	5	−0.499	−0.719	−0.278	<0.0001
	6	−0.872	−1.719	−0.026	0.0435
	7	−0.559	−1.161	0.043	0.0686
	8+	−1.502	−2.412	−0.593	0.0012
IgM^+^ or IgG^+^ or IgA^+^	1	−0.047	−0.191	0.096	0.5189
	2	0.178	−0.002	0.358	0.0521
	3	−0.202	−0.432	0.028	0.0850
	4	−0.932	−1.596	−0.267	0.0060
	5	−0.182	−0.522	0.159	0.2954
	6	−0.457	−0.735	−0.178	0.0013
	7	−0.430	−0.738	−0.123	0.0060
	8+	−0.591	−1.145	−0.036	0.0368
Ab Negative	1	−0.086	−0.263	0.091	0.3425
	2	−0.293	−0.505	−0.080	0.0069
	3	−0.210	−0.575	0.154	0.2582
	4	−0.450	−1.105	0.205	0.1783
	5	−0.126	−0.514	0.262	0.5239
	6	−0.323	−0.593	−0.053	0.0190
	7	−0.529	−0.741	−0.318	<0.0001
	8+	−0.874	−1.277	−0.470	<0.0001
**VACCINE TREATMENT GROUPS**					
Tat 7.5 μg, 3 ×	1	−0.169	−0.325	−0.012	0.0346
	2	−0.013	−0.253	0.226	0.9138
	3	0.062	−0.299	0.424	0.7347
	4	−1.363	−1.978	−0.748	<0.0001
	5	−0.455	−0.761	−0.149	0.0036
	6	−0.660	−1.014	−0.307	0.0003
	7	−0.429	−0.745	−0.114	0.0077
	8+	−0.805	−1.333	−0.277	0.0028
Tat 7.5 μg, 5 ×	1	−0.187	−0.324	−0.049	0.0077
	2	0.070	−0.136	0.275	0.5074
	3	−0.337	−0.646	−0.028	0.0327
	4	−0.611	−1.080	−0.141	0.0108
	5	−0.127	−0.456	0.202	0.4505
	6	−0.410	−0.702	−0.118	0.0059
	7	−0.554	−0.798	−0.309	<0.0001
	8+	−1.169	−1.659	−0.680	<0.0001
Tat 30 μg, 3 ×	1	−0.177	−0.378	0.024	0.0843
	2	−0.193	−0.422	0.036	0.0985
	3	−0.635	−0.929	−0.341	<0.0001
	4	−0.691	−1.127	−0.256	0.0019
	5	−0.302	−0.588	−0.017	0.0381
	6	−0.703	−1.146	−0.261	0.0019
	7	−0.800	−1.130	−0.471	<0.0001
	8+	−1.558	−2.253	−0.862	<0.0001
Tat 30 μg, 5 ×	1	−0.044	−0.309	0.221	0.7436
	2	−0.267	−0.535	0.001	0.0505
	3	−0.185	−0.453	0.083	0.1767
	4	−0.555	−1.205	0.095	0.0940
	5	−0.383	−0.614	−0.152	0.0011
	6	−0.645	−1.311	0.020	0.0573
	7	−0.446	−0.871	−0.020	0.0399
	8+	−0.746	−1.335	−0.157	0.0131
**VIRAL LOAD**					
More than 90%+visits with 0 copies	1	−0.243	−0.492	0.006	0.0556
	2	0.026	−0.276	0.327	0.8682
	3	−0.365	−0.793	0.063	0.0948
	4	−1.295	−2.013	−0.577	0.0004
	5	−0.342	−0.655	−0.029	0.0321
	6	−0.746	−1.419	−0.074	0.0297
	7	−0.708	−1.133	−0.282	0.0011
	8+	−1.649	−2.499	−0.799	0.0001
Less than 90%+ visits with 0 copies	1	−0.045	−0.130	0.040	0.2985
	2	−0.228	−0.338	−0.118	<0.0001
	3	−0.182	−0.306	−0.059	0.0038
	4	−0.315	−0.429	−0.201	<0.0001
	5	−0.291	−0.444	−0.139	0.0002
	6	−0.464	−0.617	−0.310	<0.0001
	7	−0.407	−0.563	−0.250	<0.0001
	8+	−0.490	−0.762	−0.218	0.0004
**CD8**^**+**^ **T-cell counts from baseline**		0.0003	0.0000	0.0006	0.0292
**ARV REGIMENS**					
NNRTI- or NRTI-based		−0.418	−0.609	−0.228	<0.0001
PI-based		−0.550	−0.737	−0.362	<0.0001

a Year since first immunization;

b Least square means;

c *Confidence interval*.

*Multivariate analysis of variance for repeated measures of longitudinal samples from 91 individuals*.

A statistically significant increase of CD4^+^ T-cell counts was observed at year 2 and then from year 4 to 6 post-vaccination in the subgroup of patients who developed anti-Tat Abs for all 3 classes (IgM, IgG, and IgA). A significant increase of the CD4^+^ T-cell counts was also found by year 4 to 6 post-vaccination in patients who developed only 2 anti-Tat Ab classes. In the patients who mounted only 1 Ab class (IgG or IGM or IgA), the increase of the CD4^+^ T-cell counts was significant only by year 5 and 6 post-vaccination. In the subgroup that did not develop anti-Tat Ab after vaccination, the CD4^+^ T-cell increase was, nonetheless, statistically significant from year 5 until year 8 (Table 2). As discussed later, these patients developed anti-Tat cellular responses after vaccination.

When the different vaccine regimens were taken into consideration, Tat 30 μg, 3 × was the most effective, inducing early (year 1) statistically significant and durable CD4^+^ T-cell increases, followed by 30 μg 5 ×, and 7.5 μg, 5 × that were similarly effective, but with significant increases only since year 4 or 5 (Table 2), whereas the 7.5 μg, 3 × vaccine regimen was the least effective, showing a statistically significant CD4^+^ T-cell increase only at year 5.

The evaluation of residual viremia/viral blips indicated that vaccinees with VL = 0 in more than 90% visits experienced CD4^+^ T-cell increases that became significant at years 4, 5, and 6, and were higher as compared to vaccinees with VL = 0 in <90% visits, who had, however, a significant CD4^+^ T-cell increase at year 2 and then from year 4 to 8 post-vaccination (Table 2).

Finally, no statistically significant differences were observed between PI-based and NNRTI-based regimens on CD4^+^ T-cell counts (Table 2).

Concerning HIV DNA decay, as shown in Table 3, the earliest, most pronounced, durable and statistically significant HIV-1 DNA reduction occurred in vaccinees with all or at least 2 classes of anti-Tat Abs, starting at year 1 or 3 after vaccination; in contrast, in vaccinees positive for a single anti-Tat Ab class or negative for all Ab classes the decrease was less prominent but remained statistically significant from year 6 to 8 post-vaccination (Table 3).

When the vaccine regimens were taken into consideration, Tat 30 μg, 3 × was again the most effective, showing the most pronounced and durable HIV-1 DNA decrease since year 3 post-vaccination, although vaccinees in the other regimens also showed durable, but less pronounced, and significant decreases with early decay kinetics (since year 1 or 2)(Table 3).

When residual viremia/viral blips were evaluated, vaccinees with VL = 0 in more than 90% visits experienced a pronounced HIV DNA decay starting from year 4, whereas in vaccinees with VL = 0 in <90% visits, DNA decay occurred earlier (year 2) but was much less prominent (Table 3). Moreover, CD8^+^ T-cell changes from baseline were significantly and directly related to proviral DNA variations (Table 3).

Finally, non-statistically significant differences were observed between PI-based and NNRTI-based regimens, both showing a significant and similar HIV proviral DNA reduction in association with Tat vaccination (Table 3).

## Discussion

In natural HIV infection, anti-Tat Abs are found only in a small fraction of individuals (19) but, when present, correlate with the asymptomatic phase and lower disease progression (21–24). This has suggested that a Tat vaccine could be used to induce protective immunity against HIV. In the ISS T-002 trial in Italy, immunization with Tat proved not only to induce durable anti-Tat humoral responses, but also to increase cell mediated immunity (CMI) to Tat as well as to other antigens [e.g., HIV Env, Candida, and a pool of Cytomegalovirus, Epstein-Barr and influenza virus antigens (CEF)] (15). Tat vaccinees also experienced significant CD4^+^ T-cell increases well above the level achieved with cART, significant increases of B and NK cells, and equilibration of CD4^+^ and CD8^+^ T-cell central and effector memory subsets (15), suggesting a comprehensive immune restoration. In the ensuing 3 years follow-up study, vaccinees also showed a decay of blood HIV proviral DNA reservoir, which was maximal with the 30 μg 3 × vaccine regimen and became significant for both PI- and NNRTI-based cART combinations at year 2–3 after immunization (33). While a randomized placebo group was not included in the trial, these effects were not observed in the matched, non-randomized reference group receiving only cART, which served as control according to ICH regulations (15, 33, 34). Of note, both Tat vaccinees and the control group were on cART for mean of 6 years at study entry, a period of time sufficient for cART to exert maximal effects on both CD4 T-cell increase or HIV DNA decay (45–47).

These studies have shown that HIV DNA decay in Tat vaccinees is predicted by a combination of factors, such as vaccine-induced anti-Tat Abs, including Abs capable of neutralizing HIV Env entry in DCs, and CMI against Tat (33). A subsequent randomized, placebo-controlled trial conducted in South Africa has confirmed the immunological effects of the Tat vaccine, while proviral DNA decay analysis waits for additional testing at later time points (35).

The results of the present 8-year extended follow-up study confirms the durability of specific humoral responses, which were still detectable after 8 years in about 50% of the vaccinees in the Tat 30 μg regimens, and shows a long-lasting (up to 8 years) stabilization of CD4^+^ T-cell increases, with a net average gain of about 100 cells/μL. Of note, CD4^+^ T-cell count restoration occurred even in subjects with a CD4^+^ T-cell nadir ≤250 cells/μL and, as by the quartile regression analysis, even in cART poor immunological responders (i.e., <500 CD4^+^ T-cells/μL at baseline), who are known to experience disease progression and comorbidities (25, 29, 48, 49).

A striking finding of this study is represented by the continued HIV DNA decay that, in vaccinees of the 30 μg 3 × regimen, was reduced to 10% of its pre-vaccination size. This, in fact, conflicts with the known stabilization of HIV DNA after about 4 years of cART into a residual, steady reservoir core (50, 51), as also observed by us in the reference control group of our trial (33). Notably, the rate kinetics of HIV DNA decay in Tat vaccinees appear to be generally much faster as compared to patients treated for comparable period of times. In fact, in two recent studies evaluating DNA decay kinetics in patients on long-term cART, the HIV DNA half-life was quantitated in 7–19 years (52, 53), as compared to only 3 years for Tat vaccinees (Figure 5). One of these studies also evaluated the relationship between HIV DNA decay and intermittent episodes of plasma viremia, as we have done in our study since VL is a factor known to be associated with slower rates of decay (45–47). Notably, the kinetics of DNA decay appear remarkably and consistently faster for Tat vaccinees, irrespective of intermittent viremia, with half-life values of 1, 3, or 4 years for Tat vaccinees with VL = 0, VL = 1–40 or VL >40 copies/mL, respectively (Figure 6B), as compared to 7, 12, or 22 years for cART-treated patients in the absence of vaccination (52). These data suggest that Tat immunization accelerates latent HIV reservoir decay in cART-treated patients.

Most notably, those vaccinees fully virologically suppressed at all visits throughout the 8-year follow-up showed a time to eradication of 31 years (Figure 6B). Although this subgroup was characterized by a small sample size (*n* = 4), these data advocate to explore whether immunization with Tat may lead to “within-life span” virus eradication in cART fully-suppressed patients. On the other hand, the data also suggest that poorly compliant patients may also experience a proviral DNA decay and containment of CD4^+^ T-cell count decline and VL increase after Tat vaccination, as also indicated by our trial in South Africa in patients not compliant to therapy (35).

The analysis of the factors predictive of CD4^+^ T-cell gains and proviral DNA decay suffers in this study from the lack of continuous measurement of all the parameters previously shown to participate in vaccine-induced immunity, including, in particular, anti-Tat Abs neutralizing HIV entry in DCs and CMI to Tat, which was not possible to monitor throughout the whole 8 years period. In fact, as evidenced by the multivariate regression analysis, anti-Tat Abs measured by ELISA were not fully predictive, as also anti-Tat Ab-negative vaccinees showed significant CD4^+^ T-cell increases and proviral DNA decay rates, although at later periods of time and to a lower extent. However, 81% of anti-Tat Ab-negative vaccinees developed in the meantime CMI to Tat (last measurement: week 144, data not shown). This suggests that CMI may contribute to DNA decay also in the long-term as previously observed in the short-term (3 years post-vaccination) (33), in substantial agreement with the protective role of CMI and, in particular, of anti-Tat cytotoxic T lymphocytes (CTLs) previously evidenced in Tat vaccinated monkeys (13).

The Tat vaccinees experienced an increase of the CD4^+^/CD8^+^ T-cell ratio, an important marker of immune reconstitution in virologically suppressed cART-treated patients (54), which was observed in 33/57 (58%) vaccinees with levels below 1 at study entry. An inverse relationship between the size of virus reservoir and CD4^+^/CD8^+^ T-cell ratio has been reported for patients responding to cART (55–58), and this relation is indeed confirmed by our study. In fact, the increase of the ratio above cART levels upon Tat vaccination was paralleled by a continued, long-lasting decay of HIV-1 proviral DNA throughout the 8 years of follow-up. Notably, proviral decay was more pronounced in vaccinees experiencing CD4^+^/CD8^+^ T-cell ratio or CD4^+^ T-cell increases, while it was less pronounced in vaccinees with increasing CD8^+^ T-cell counts. Further, although CD4^+^ T-cell increases plateaued after year 5, proviral DNA continued to decay. In their whole, these data suggest that continued, long-term control of the virus reservoirs is related to the amelioration of immune homeostasis induced by the Tat vaccine.

CD4^+^ T cells transitioning from the effector to the resting memory state were recently shown to be particularly susceptible to latent HIV infection (59). It was also reported that stimulation of HIV-specific CTLs favors killing of latently infected CD4^+^ T-cells during HIV reactivation (60). Further, it has been shown that residual HIV replication in lymphoid tissues with suboptimal antiviral drug penetration can replenish the reservoirs in cART-treated patients (61). Conversely, extracellular Tat is known to favor the differentiation of naïve CD4^+^ T-cells toward the effector-memory phenotype (62), and to inhibit the activation of CD8^+^ T-cells (63). These data advocate studies to verify whether anti-Tat Abs may block Tat-induced CD4^+^ T-cell transitioning through a functional cell state primed for latent HIV infection and/or relieving Tat-mediated inhibition of CTL responses.

Overall the findings reported herein indicate that anti-Tat immune responses can compensate for cART shortfalls and promote return to immune homeostasis, which likely contributes to restoration of effective antiviral responses that, together with anti-Tat immunity, are capable of attacking cART-resistant virus reservoirs. Thus, Tat immunization represents a promising pathogenesis-driven intervention to intensify cART efficacy while renewing perspectives for a functional cure. Future therapy interruption studies will provide proof-of-concept that Tat vaccine recipients (particularly those with high titers and all classes of anti-Tat Ab responses) may be able to stay off therapy safely for periods of time to be determined in *ad hoc* trials.

## Author Contributions

CS and PM contributed equally to the manuscript preparation, supervised the experimental work, data analysis and interpretation. CS supervised the clinical study management. AT coordinated the experimental work and contributed to data and manuscript preparation. OP contributed to the clinical study management, performed the statistical analyses and contributed to manuscript preparation. AnC and CO performed HIV-1 DNA quantification. SM contributed to the clinical study management and manuscript preparation. VF, AA, GP, and MC performed the experimental work. SB performed part of the statistical analyses. MrM, SN, LS, AL, AM, AS, MDP, and MG conducted the study at the clinical sites. AuC contributed to data and manuscript critical review. MuM supervised the HIV-1 DNA evaluation and contributed to data and manuscript critical review. FE supervised the experimental work and contributed to data and manuscript critical review. BE conceived and designed the study, supervised the experimental work, data analysis and interpretation, and manuscript preparation.

### Conflict of Interest Statement

MuM hold shares in Diatheva SrL. The remaining authors declare that the research was conducted in the absence of any commercial or financial relationships that could be construed as a potential conflict of interest.

## Supplementary Material

Supplementary Figure 1 Flow-chart for the ISS T-002 and ISS T-002 EF-UP studies. One hundred sixty-eight HIV-infected cART-treated volunteers were enrolled in the ISS T-002 trial and randomly allocated to one of the four treatment groups to receive intradermal injections of biologically active HIV-1 Tat protein at the indicated doses without any adjuvant 1 month apart. All participants were analyzed for safety and 155 for immunogenicity (15, 33). Ninety-three subjects of the immunogenicity population were enrolled in the ISS T-002 EF-UP study. One subject was excluded from the analyses due to non-compliance to therapy, therefore 92 subjects were analyzed.

Supplementary Figure 2 Anti-Tat Ab persistence by the number of anti-Tat Ab classes over 8 years of follow-up. Kaplan-Meier estimates showing the cumulative probability of anti-Tat Ab durability in ISS T-002 responders (*n* = 71) stratified according to the number of anti-Tat Ab classes induced by vaccination (1, 2, or 3 classes) up to 412 weeks of follow-up (median follow-up: 316 weeks).

Supplementary Figure 3 Changes over baseline of CD4^+^ T-cells stratified by CD4^+^ T-cell nadir during 8 years of follow-up. Baseline values (left panels) and annual changes over baseline (right panels) from ISS T-002 study entry of CD4^+^ T cells stratified by CD4^+^ T-cell nadir are shown. Vaccinees with CD4^+^ T-cell nadir ≤ 250 cells/μL: *n* = 20, >250 cells/μL: *n* = 72. Data are presented as mean values with standard error. A longitudinal analysis for repeated measurements was applied. *p*-values assess the values at year 1–8 after immunization vs. baseline values.

Supplementary Figure 4 Longitudinal regression analysis of CD4^+^ T-cells stratified by Tat vaccine regimens. Tat vaccine dose-effects on the increase of CD4^+^ T cells by days. A longitudinal analysis using a random-effects regression model was performed.

Supplementary Figure 5 Changes of blood HIV-1 proviral DNA load over baseline in vaccinees stratified by cART regimen during follow-up. Baseline values (left panels) and annual changes (right panels) of HIV DNA levels (expressed as log_10_ copies/10^6^ CD4^+^ T-cells) from ISS T-002 study entry in vaccinees stratified by cART regimen are shown. The number of participants tested are as follows: year 1 *n* = 89, year 2 *n* = 59, year 3 *n* = 42, year 4 *n* = 36, year 5 *n* = 51, year 6 *n* = 75, year 7 *n* = 58, year 8+ *n* = 37. Data are presented as mean values with standard error. A longitudinal analysis for repeated measurements was applied. *p*-values assess the values at year 1–8 after immunization vs. baseline values.

Supplementary Figure 6 Longitudinal regression analysis of HIV-1 proviral DNA decay in vaccinees stratified by vaccine regimens. Tat vaccine dose-effects on the proviral DNA decay (expressed as log_10_ copies/10^6^ CD4^+^ T-cells) by a longitudinal analysis using a random-effects regression model is shown.

Supplementary Figure 7 Variations upon time of CD4^+^ T-cell number stratified according to baseline CD4^+^ T-cell quartiles. Linear regression mixed effect model for variations upon time of CD4^+^ T-cell number stratified by baseline CD4^+^ T-cell quartiles. CD4^+^ T-cell quartiles at baseline: Q1 <493 (*n* = 23), Q2 493–600 (*n* = 24), Q3 601–734 (*n* = 22) and Q4 >734 (*n* = 22). Y-axis shows predicted values.

Supplementary Figure 8 Variations upon time of HIV-1 proviral DNA stratified according to baseline HIV-1 proviral DNA quartiles. Linear regression mixed effect model for variations upon time of HIV-1 proviral DNA (log_10_ copies/10^6^ CD4^+^ T-cells) stratified by baseline HIV-1 proviral DNA quartiles. HIV-1 proviral DNA quartiles at baseline: Q1 <2.86 (*n* = 22), Q2 2.86–3.10 (*n* = 24), Q3 3.11–3.47 (*n* = 23) and Q4 >3.47 (*n* = 22). Y-axis shows predicted values.

Supplementary Figure 9 Relationship of CD4^+^ T-cells **(A)**, CD8^+^ T-cells **(B)** and HIV-1 proviral DNA in vaccinees during follow-up. Relationships between changes of HIV proviral DNA levels from baseline (log_10_ copies/10^6^ CD4^+^ T-cells) and the changes of CD4^+^ T-cells **(A)** or CD8^+^ T-cells **(B)** from baseline are shown. A generalized estimating equation with adjustment for repeated measures was utilized.
