# Supplementary figures and images for: Continued Decay of HIV Proviral DNA Upon Vaccination With HIV-1 Tat of Subjects on Long-Term ART: An 8-Year Follow-Up Study
Source: Front Immunol. 2019 Feb 13;10:233. doi: 10.3389/fimmu.2019.00233 (PMC6381398; doi:10.3389/fimmu.2019.00233)

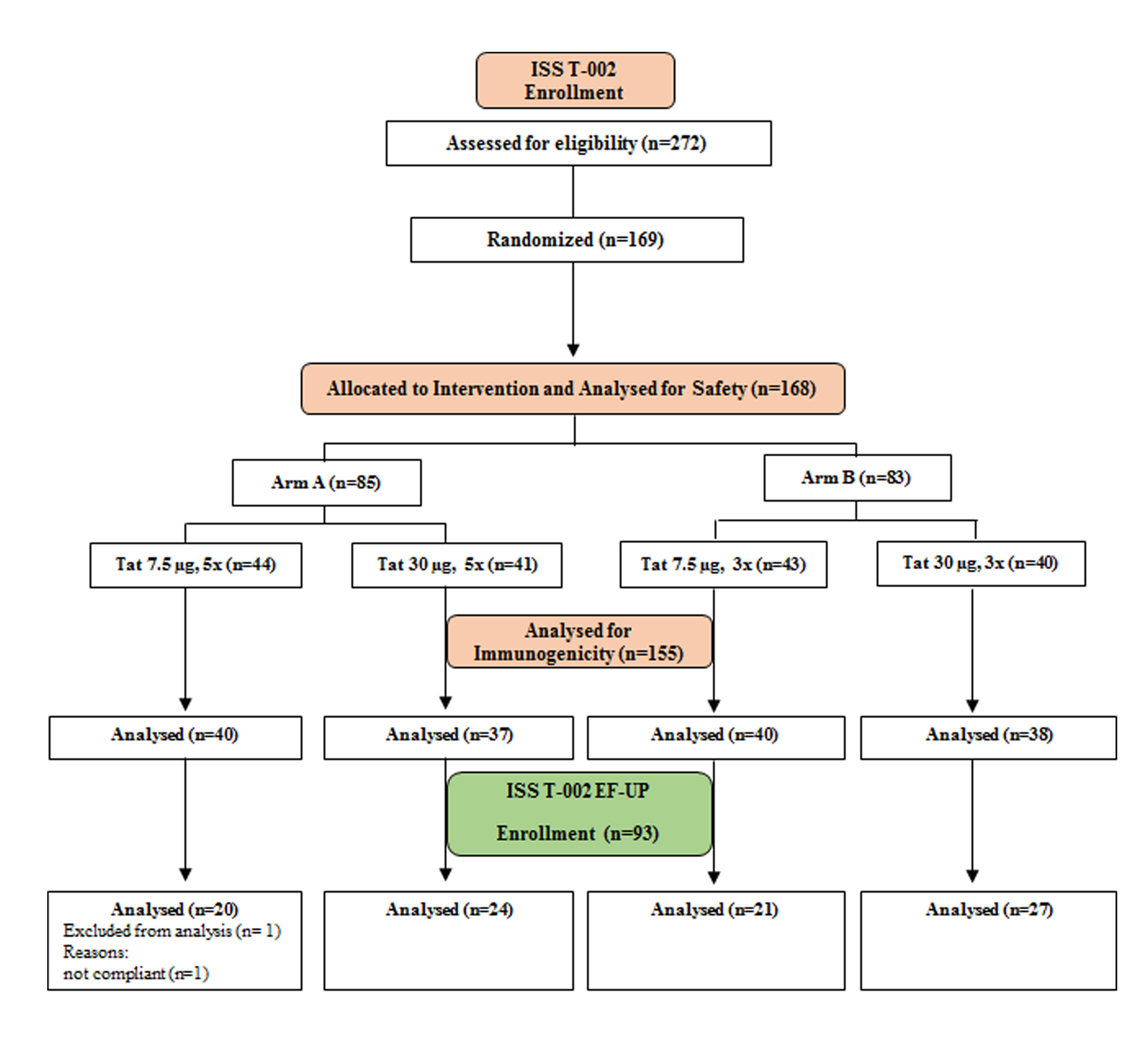

Supplement: Supplementary Figure 1 — Flow-chart for the ISS T-002 and ISS T-002 EF-UP studies. One hundred sixty-eight HIV-infected cART-treated volunteers were enrolled in the ISS T-002 trial and randomly allocated to one of the four treatment groups to receive intradermal injections of biologically active HIV-1 Tat protein at the indicated doses without any adjuvant 1 month apart. All participants were analyzed for safety and 155 for immunogenicity (15, 33). Ninety-three subjects of the immunogenicity population were enrolled in the ISS T-002 EF-UP study. One subject was excluded from the analyses due to non-compliance to therapy, therefore 92 subjects were analyzed. [file Image_1.JPEG]

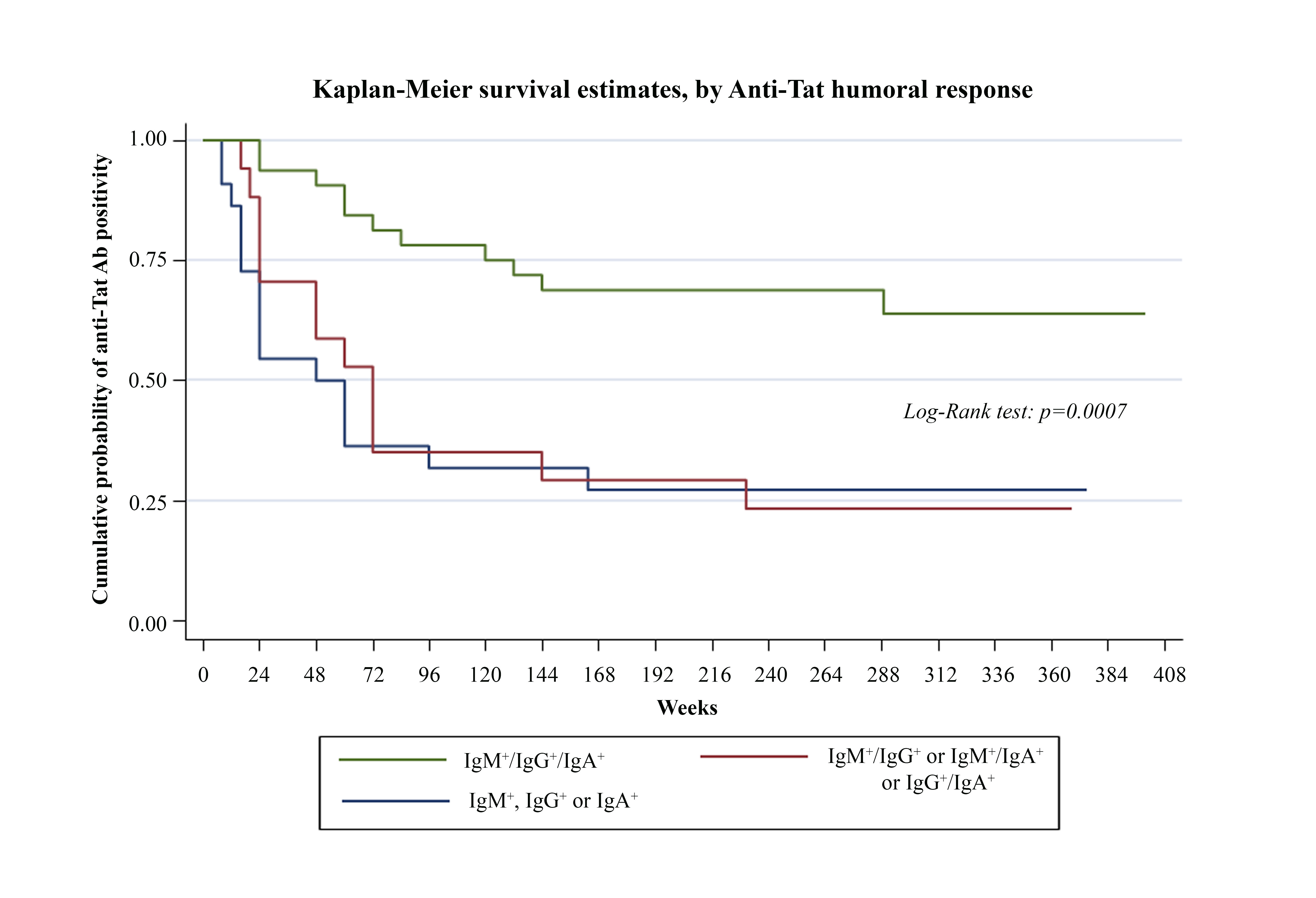

Supplement: Supplementary Figure 2 — Anti-Tat Ab persistence by the number of anti-Tat Ab classes over 8 years of follow-up. Kaplan-Meier estimates showing the cumulative probability of anti-Tat Ab durability in ISS T-002 responders (n = 71) stratified according to the number of anti-Tat Ab classes induced by vaccination (1, 2, or 3 classes) up to 412 weeks of follow-up (median follow-up: 316 weeks). [file Image_2.JPEG]

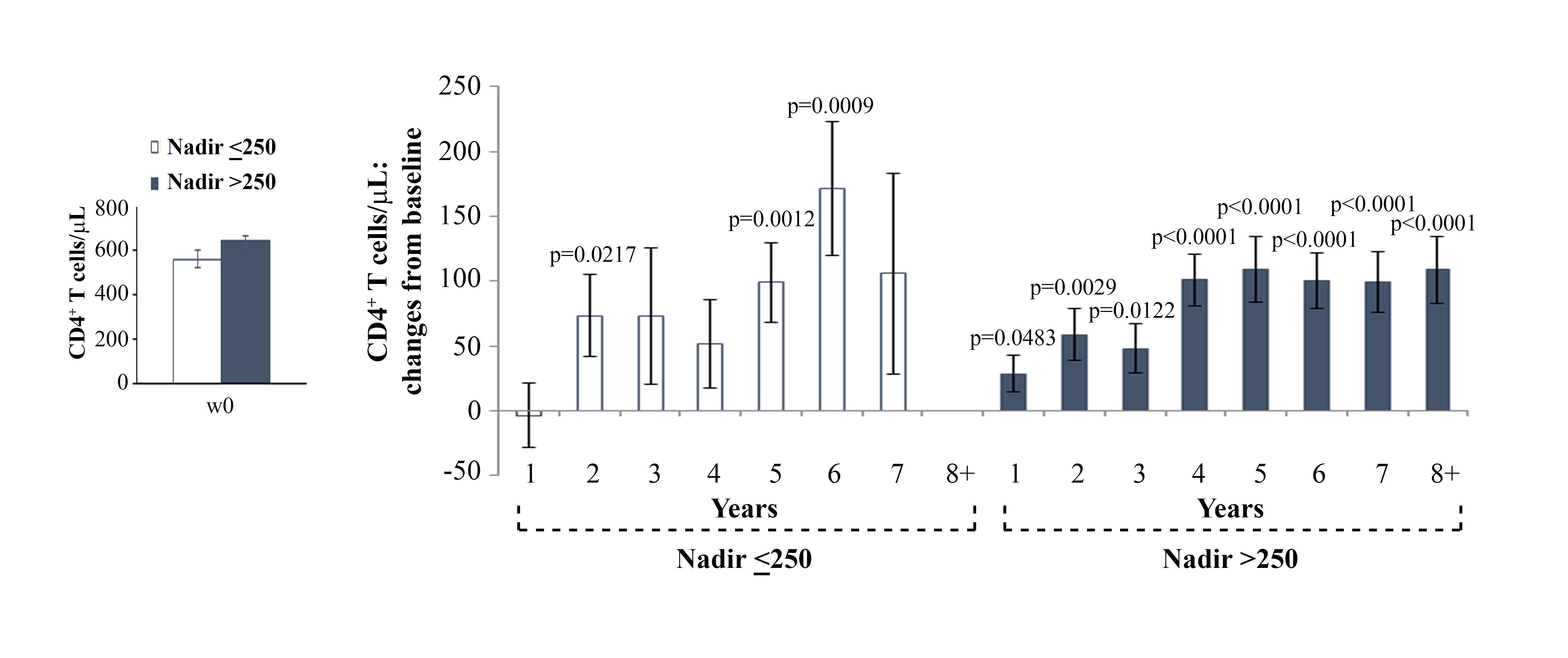

Supplement: Supplementary Figure 3 — Changes over baseline of CD4+ T-cells stratified by CD4+ T-cell nadir during 8 years of follow-up. Baseline values (left panels) and annual changes over baseline (right panels) from ISS T-002 study entry of CD4+ T cells stratified by CD4+ T-cell nadir are shown. Vaccinees with CD4+ T-cell nadir ≤ 250 cells/μL: n = 20, >250 cells/μL: n = 72. Data are presented as mean values with standard error. A longitudinal analysis for repeated measurements was applied. p-values assess the values at year 1–8 after immunization vs. baseline values. [file Image_3.JPEG]

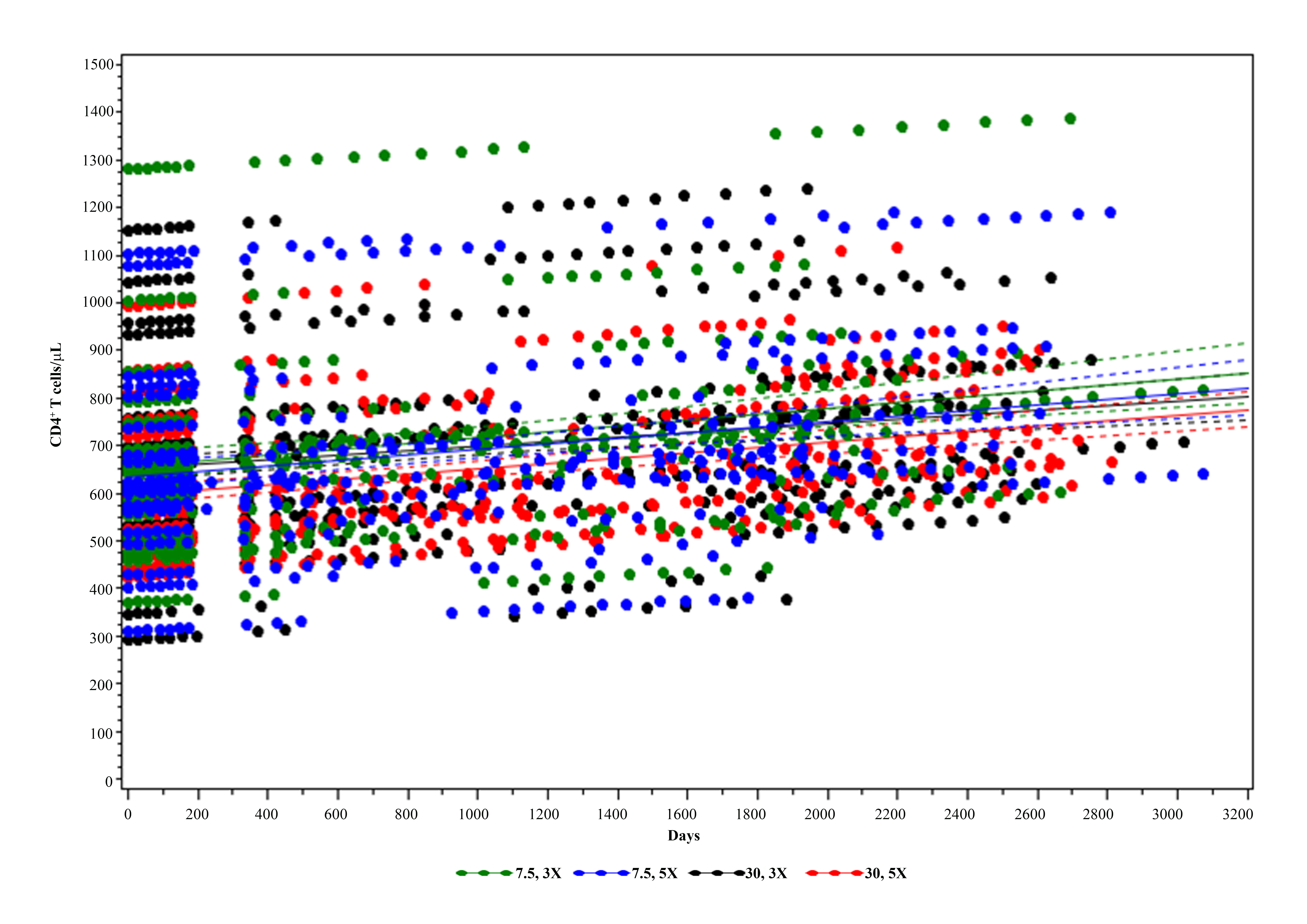

Supplement: Supplementary Figure 4 — Longitudinal regression analysis of CD4+ T-cells stratified by Tat vaccine regimens. Tat vaccine dose-effects on the increase of CD4+ T cells by days. A longitudinal analysis using a random-effects regression model was performed. [file Image_4.JPEG]

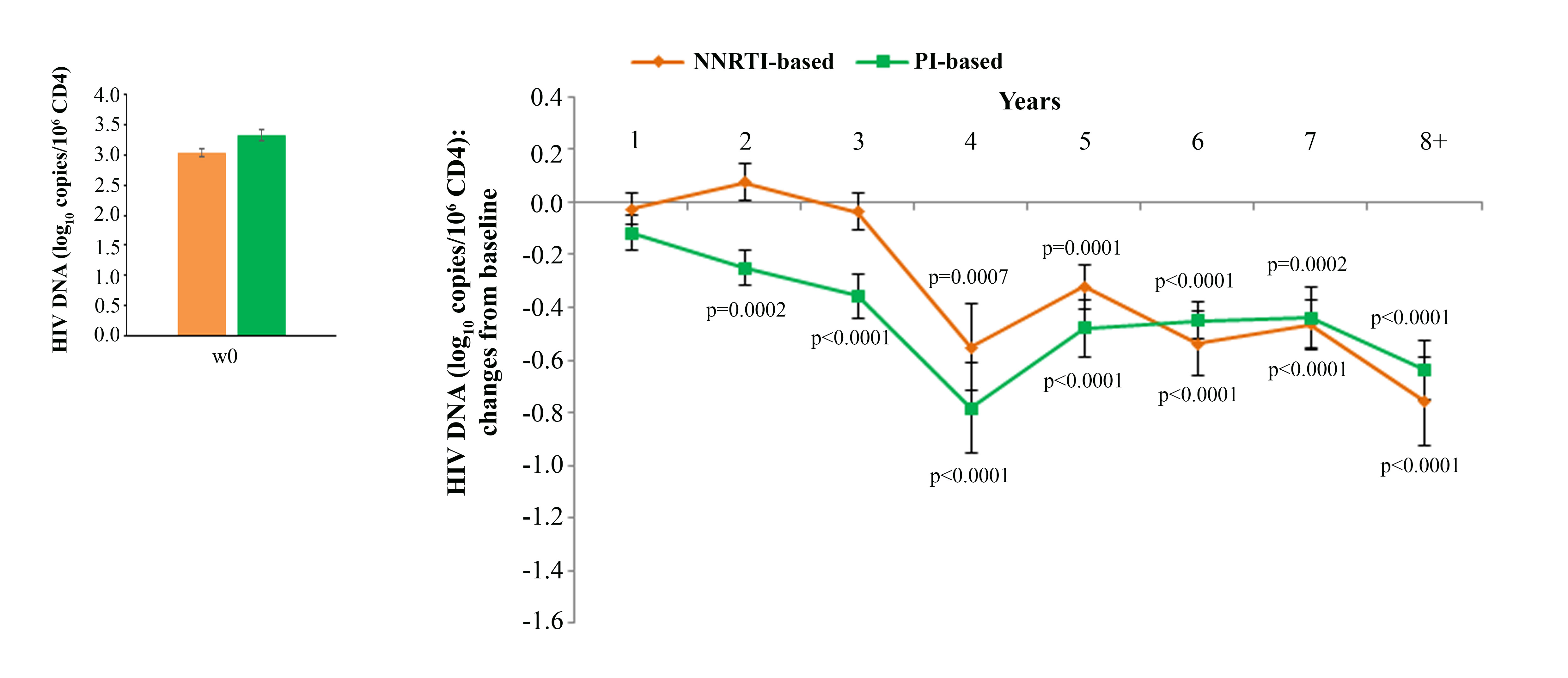

Supplement: Supplementary Figure 5 — Changes of blood HIV-1 proviral DNA load over baseline in vaccinees stratified by cART regimen during follow-up. Baseline values (left panels) and annual changes (right panels) of HIV DNA levels (expressed as log10 copies/106 CD4+ T-cells) from ISS T-002 study entry in vaccinees stratified by cART regimen are shown. The number of participants tested are as follows: year 1 n = 89, year 2 n = 59, year 3 n = 42, year 4 n = 36, year 5 n = 51, year 6 n = 75, year 7 n = 58, year 8+ n = 37. Data are presented as mean values with standard error. A longitudinal analysis for repeated measurements was applied. p-values assess the values at year 1–8 after immunization vs. baseline values. [file Image_5.JPEG]

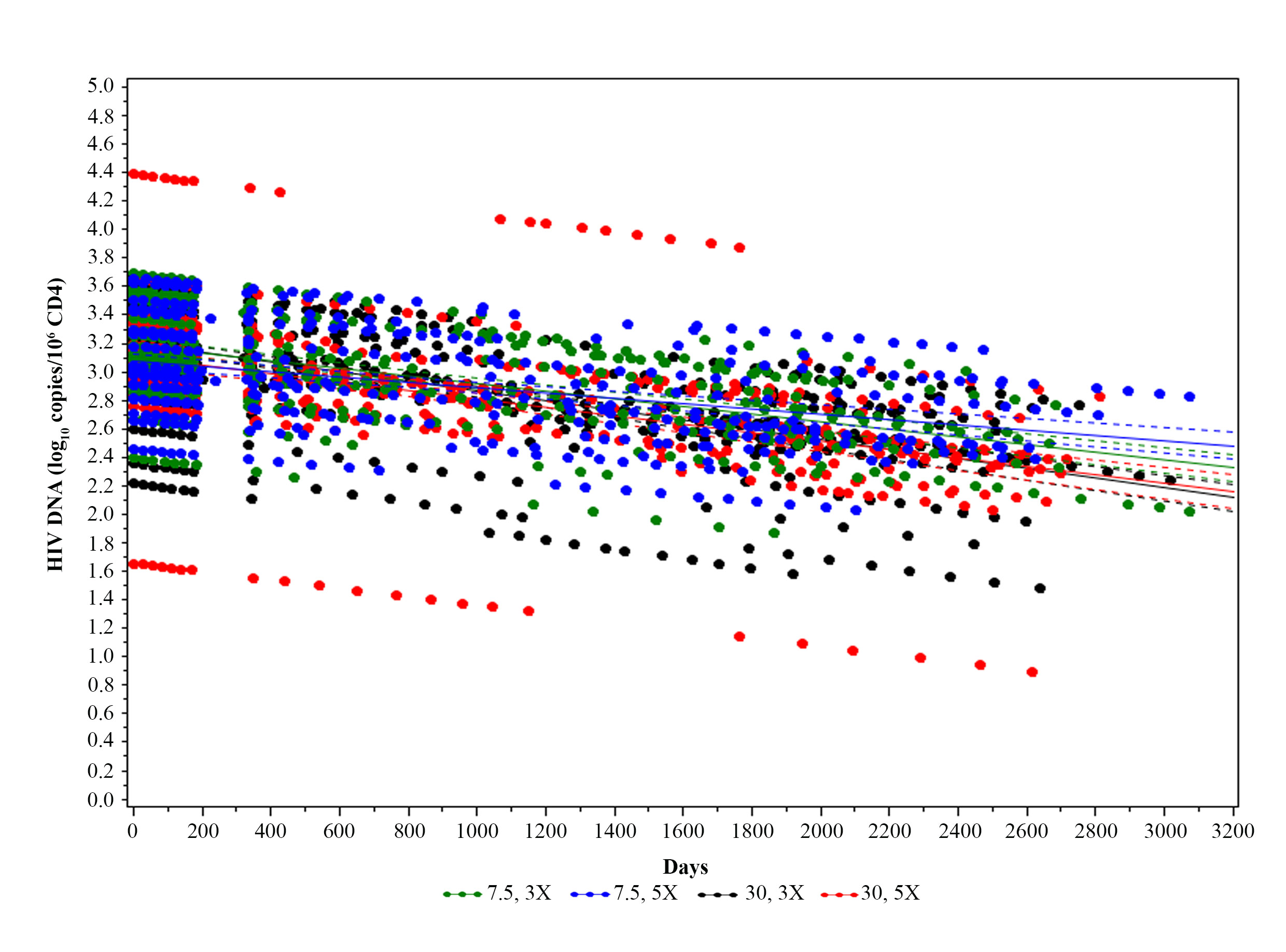

Supplement: Supplementary Figure 6 — Longitudinal regression analysis of HIV-1 proviral DNA decay in vaccinees stratified by vaccine regimens. Tat vaccine dose-effects on the proviral DNA decay (expressed as log10 copies/106 CD4+ T-cells) by a longitudinal analysis using a random-effects regression model is shown. [file Image_6.JPEG]

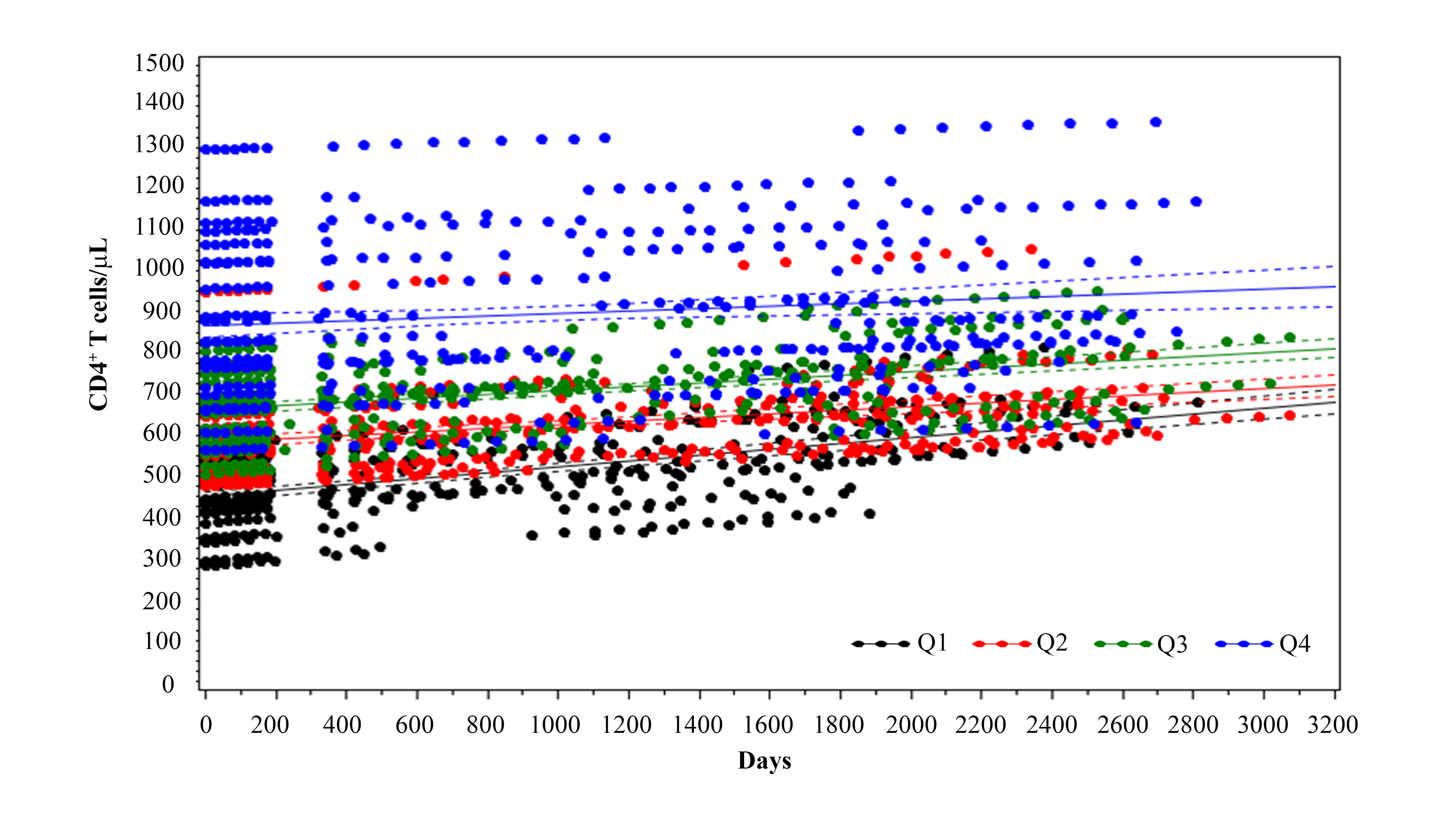

Supplement: Supplementary Figure 7 — Variations upon time of CD4+ T-cell number stratified according to baseline CD4+ T-cell quartiles. Linear regression mixed effect model for variations upon time of CD4+ T-cell number stratified by baseline CD4+ T-cell quartiles. CD4+ T-cell quartiles at baseline: Q1 <493 (n = 23), Q2 493–600 (n = 24), Q3 601–734 (n = 22) and Q4 >734 (n = 22). Y-axis shows predicted values. [file Image_7.JPEG]

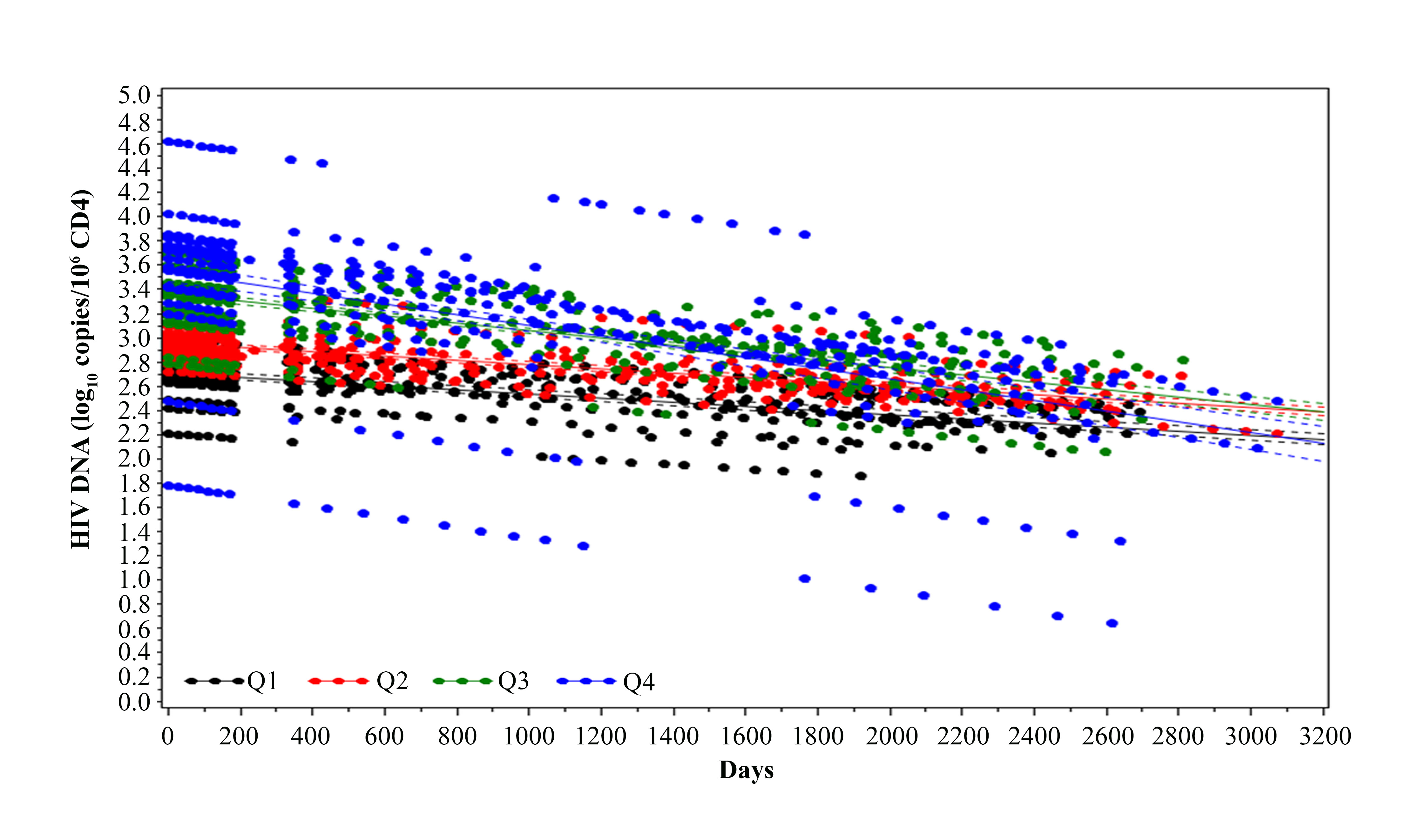

Supplement: Supplementary Figure 8 — Variations upon time of HIV-1 proviral DNA stratified according to baseline HIV-1 proviral DNA quartiles. Linear regression mixed effect model for variations upon time of HIV-1 proviral DNA (log10 copies/106 CD4+ T-cells) stratified by baseline HIV-1 proviral DNA quartiles. HIV-1 proviral DNA quartiles at baseline: Q1 <2.86 (n = 22), Q2 2.86–3.10 (n = 24), Q3 3.11–3.47 (n = 23) and Q4 >3.47 (n = 22). Y-axis shows predicted values. [file Image_8.JPEG]

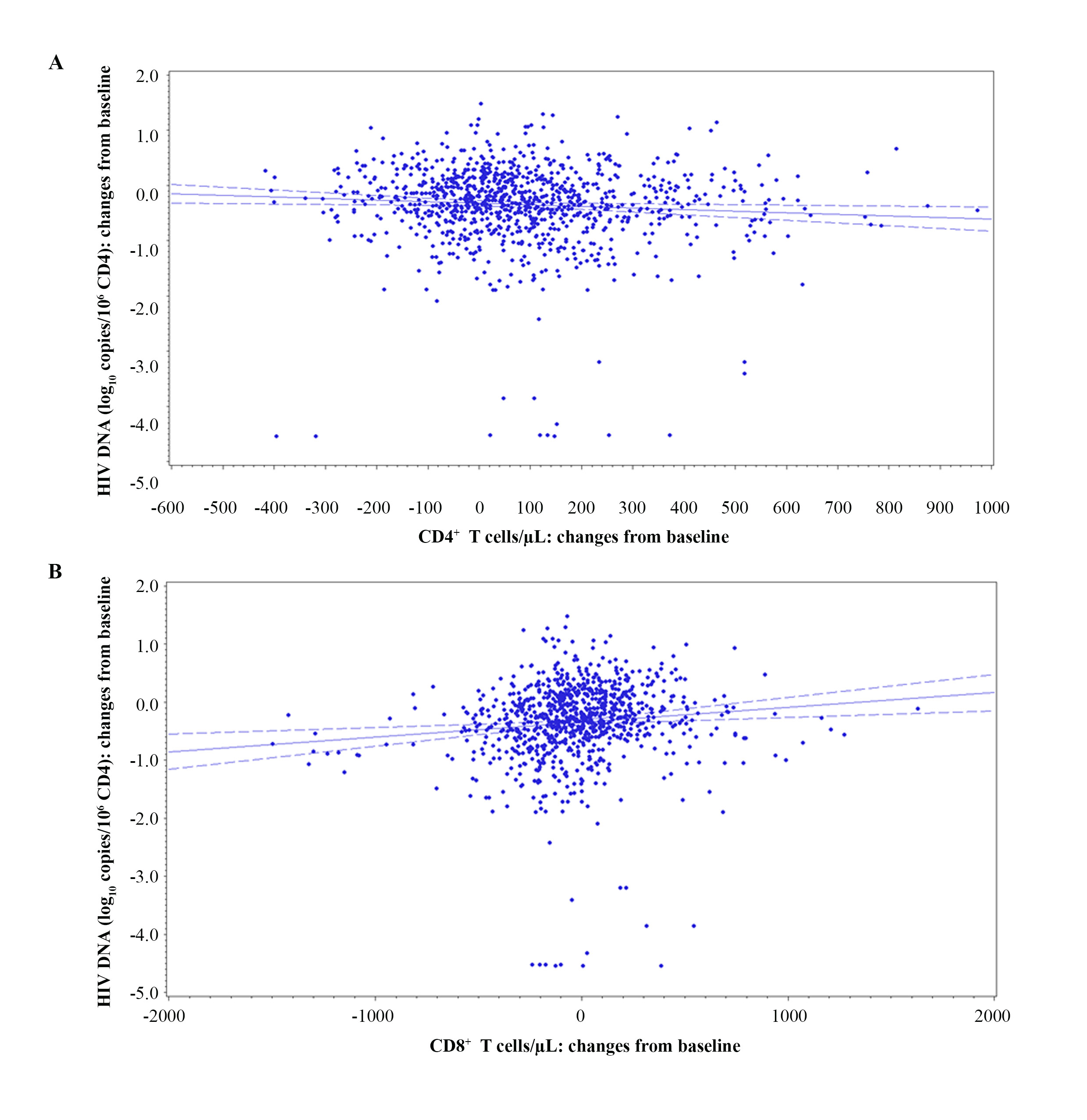

Supplement: Supplementary Figure 9 — Relationship of CD4+ T-cells (A), CD8+ T-cells (B) and HIV-1 proviral DNA in vaccinees during follow-up. Relationships between changes of HIV proviral DNA levels from baseline (log10 copies/106 CD4+ T-cells) and the changes of CD4+ T-cells (A) or CD8+ T-cells (B) from baseline are shown. A generalized estimating equation with adjustment for repeated measures was utilized. [file Image_9.JPEG]
