# Supplementary material for: Continued Decay of HIV Proviral DNA Upon Vaccination With HIV-1 Tat of Subjects on Long-Term ART: An 8-Year Follow-Up Study
Source: Front Immunol. 2019 Feb 13;10:233. doi: 10.3389/fimmu.2019.00233 (PMC6381398; doi:10.3389/fimmu.2019.00233)
Supplement: Supplementary file 10 [file Table_1.DOCX]

**Supplementary Table 1: Distribution of vaccinees by frequency of visits with viral load (VL)=0 and by VL classes**

| **Frequency** |  | | | | | |
| --- | --- | --- | --- | --- | --- | --- |
| **Percent** | **%visits with VL =0** | **VL classes (copies/mL)*** | | | | |
| **Row Pct** |  | **0** | **1-40** | **41-99** | **100+** | **Total** |
| **Col Pct** | **90%+** | 4 | 13 | 2 | 0 | 19 |
|  |  | 4.35 | 14.13 | 2.17 | 0 | 20.65 |
|  |  | 21.05 | 68.42 | 10.53 | 0 |  |
|  |  | 100 | 26 | 8.33 | 0 |  |
|  | **70%-89%** | 0 | 24 | 7 | 2 | 33 |
|  |  | 0 | 26.09 | 7.61 | 2.17 | 35.87 |
|  |  | 0 | 72.73 | 21.21 | 6.06 |  |
|  |  | 0 | 48 | 29.17 | 14.29 |  |
|  | **50%-69%** | 0 | 9 | 6 | 5 | 20 |
|  |  | 0 | 9.78 | 6.52 | 5.43 | 21.74 |
|  |  | 0 | 45 | 30 | 25 |  |
|  |  | 0 | 18 | 25 | 35.71 |  |
|  | **<50%** | 0 | 4 | 9 | 7 | 20 |
|  |  | 0 | 4.35 | 9.78 | 7.61 | 21.74 |
|  |  | 0 | 20 | 45 | 35 |  |
|  |  | 0 | 8 | 37.5 | 50 |  |
|  | **Total** | 4 | 50 | 24 | 14 | 92 |
|  |  | 4.35 | 54.35 | 26.09 | 15.22 | 100 |

* For inclusion in the VL classes the highest VL reached during the whole study period was considered.
