# Supplementary material for: Continued Decay of HIV Proviral DNA Upon Vaccination With HIV-1 Tat of Subjects on Long-Term ART: An 8-Year Follow-Up Study
Source: Front Immunol. 2019 Feb 13;10:233. doi: 10.3389/fimmu.2019.00233 (PMC6381398; doi:10.3389/fimmu.2019.00233)
Supplement: Supplementary file 11 [file Data_Sheet_1.PDF]

Supplementary Data to Figures. Linear regression parameters and p-values

Supplementary Figure 4

**Tat vaccine regimens**	**Patients**	**Parameter***	**p-value****
Tat 30 µg, 5x	24	β = +0.06/day	<0.0001
Tat 30 µg, 3x	27	β = +0.05/day	<0.0001
Tat 7.5 µg, 5x	20	β = +0.04/day	<0.0001
Tat 7.5 µg, 3x	21	β = +0.04/day	<0.0001

Supplementary Figure 6

**Tat vaccine regimens**	**Patients**	**Parameter***	**p-value****
Tat 30 µg, 3x	25	β = -0.00035/day	<0.0001
Tat 7.5 µg, 3x	20	β = -0.00030/day	<0.0001
Tat 30 µg, 5x	24	β = -0.00028/day	<0.0001
Tat 7.5 µg, 5x	20	β = -0.00019/day	<0.0001

Figure 6A

**VL Frequency Class**	**Patients**	**Parameter***	**p-value****
VL=0 in more than 90% of visits	19	β = -0.00043/day	<0.0001
VL=0 in less than 90% of visits	73	β = -0.00024/day	<0.0001

Figure 6B

**VL Cut-off Class**	**Patients**	**Parameter***	**p-value****
0 RNA copies/mL	4	β = -0.00076/day	<0.0001
1-40 RNA copies/mL	50	β = -0.00024/day	<0.0001
41-99 RNA copies/mL	24	β = -0.00027/day	<0.0001
>100 RNA copies/mL	14	β = -0.00023/day	<0.0001

Supplementary Figure 7

**$CD4^+$ T-cell quartiles**	**Patients**	**Parameter***	**p-value****
Q1	23	β = +0.07/day	<0.0001
Q2	24	β = +0.04/day	<0.0001
Q3	22	β = +0.05/day	<0.0001
Q4	22	β = +0.03/day	<0.0001

Supplementary Figure 8

**HIV-1 proviral DNA quartiles**	**Patients**	**Parameter***	**p-value****
Q1	22	β = -0.00018 /day	<0.0001
Q2	24	β = -0.00018/day	=0.0004
Q3	23	β = -0.00030/day	<0.0001
Q4	22	β = -0.00044/day	<0.0001

*Decay slope (or rate constant) assuming a first-order kinetics
**Probability of slope being not different from zero
